# 7-(2-Anilinopyrimidin-4-yl)-1-benzazepin-2-ones Designed by a “Cut and Glue” Strategy Are Dual Aurora A/VEGF-R Kinase Inhibitors

**DOI:** 10.3390/molecules26061611

**Published:** 2021-03-14

**Authors:** Mehmet Karatas, Apirat Chaikuad, Bianca Berger, Michael H. G. Kubbutat, Frank Totzke, Stefan Knapp, Conrad Kunick

**Affiliations:** 1 Institut für Medizinische und Pharmazeutische Chemie, Technische Universität Braunschweig, Beethovenstraße 55, 38106 Braunschweig, Germany; mehmetkaratas02@hotmail.de (M.K.); Bianca_Berger@web.de (B.B.); 2 Zentrum für Pharmaverfahrenstechnik (PVZ), Technische Universität Braunschweig, Franz-Liszt-Straße 35A, 38106 Braunschweig, Germany; 3 Structural Genomics Consortium, BMLS, Max-von-Laue-Straße 15, 60438 Frankfurt am Main, Germany; chaikuad@pharmchem.uni-frankfurt.de (A.C.); knapp@pharmchem.uni-frankfurt.de (S.K.); 4 Institut für Pharmazeutische Chemie, Johann Wolfgang-Goethe-Universität, Max-von-Laue-Straße 9, 60438 Frankfurt am Main, Germany; 5 Reaction Biology Europe GmbH, 79108 Freiburg, Germany; M.Kubbutat@reactionbiology.de (M.H.G.K.); F.Totzke@reactionbiology.de (F.T.)

**Keywords:** anilinopyrimidine, Aurora kinase, benzazepinone, molecular docking, protein kinase inhibitor, sulfamide, X-ray structure analysis

## Abstract

Although overexpression and hyperactivity of protein kinases are causative for a wide range of human cancers, protein kinase inhibitors currently approved as cancer drugs address only a limited number of these enzymes. To identify new chemotypes addressing alternative protein kinases, the basic structure of a known PLK1/VEGF-R2 inhibitor class was formally dissected and reassembled. The resulting 7-(2-anilinopyrimidin-4-yl)-1-benzazepin-2-ones were synthesized and proved to be dual inhibitors of Aurora A kinase and VEGF receptor kinases. Crystal structures of two representatives of the new chemotype in complex with Aurora A showed the ligand orientation in the ATP binding pocket and provided the basis for rational structural modifications. Congeners with attached sulfamide substituents retained Aurora A inhibitory activity. In vitro screening of two members of the new kinase inhibitor family against the cancer cell line panel of the National Cancer Institute (NCI) showed antiproliferative activity in the single-digit micromolar concentration range in the majority of the cell lines.

## 1. Introduction

**Figure 1:**
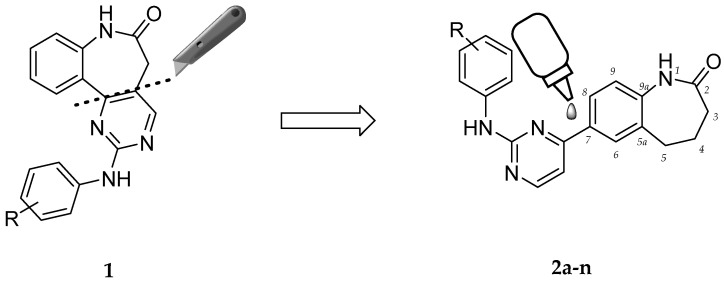
“Cut and glue” strategy: the 1-benzazepin-2-one and the anilinopyrimidine parts of the PLK-inhibitor scaffold **1** were detached at the annulation site and reconnected at the 7- and the 4-positions of the ring systems, resulting in the title structures **2a**–**n**. For residues R refer to Table 3.

Hyperactivity of protein kinases is connected to various human tumors and frequently contributes to the uncontrolled cell growth found in cancer diseases. Thus, more than forty protein kinase inhibitors have been introduced within the recent two decades as anticancer drugs, some of them with remarkable success [1,2,3]. However, these drugs already address just a small fraction of the various cancer-related protein kinases, and therefore the search for new chemotypes acting as inhibitors of these enzymes is still a major research topic in medicinal chemistry [1]. Pyrimido[5,4-*d*][1]benzazepin-6-ones **1** are a class of potent dual inhibitors of the polo-like kinase 1 (PLK1) and the vascular endothelial growth factor receptor kinase 2 (VEGF-R2) with antiproliferative activity against cancer cell lines [4]. Obviously, molecules such as **1** comprise structural features that are favorable for interactions with the ATP binding pocket of protein kinases, namely hydrogen bond donor and acceptor groups like the anilinopyrimidine and the benzazepinone elements [2,5]. We considered a rearrangement of these structural features with the goal to generate new chemical matter with protein kinase inhibitory activity. Thus, we applied a “cut and glue” strategy by dissecting the benzazepinone and the pyrimidine ring systems and reconnecting them by a covalent bond between the respective 7- and the 4-position [6,7]. The so designed structure **2a** contains no further substituents (R = H) and was therefore considered as prototype within the series of similar compounds (Figure 1). Evaluation of **2a** in a panel of 24 cancer-related protein kinases revealed that Aurora A kinase and the receptor kinases VEGF-R2 and VEGF-R3 were inhibited with IC_50_ values below 10 µM. Further structural modifications of this scaffold were directed to improve the potency regarding Aurora A. Concomitant inhibition of the VEGF receptor kinases was considered desirable, since various established multiple kinase inhibitors applied as anticancer drugs also act on this target [8], e.g., sorafenib (IC_50_ VEGF-R2 = 90 nM) [9], or cabozantinib (IC_50_ VEGF-R2 = 0.035 nM) [10].

**Figure 2:**
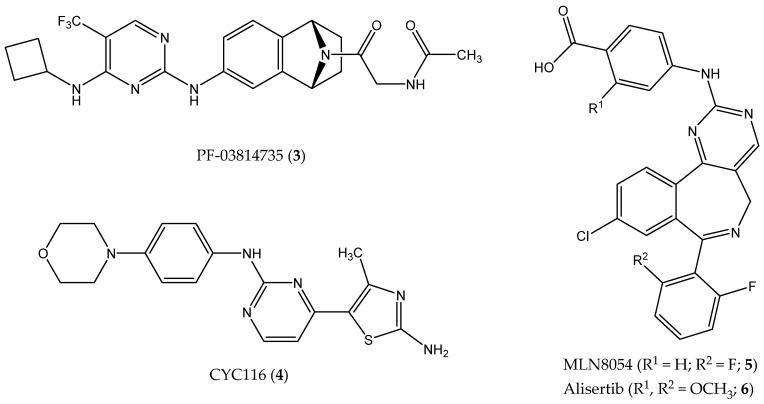
Aurora kinase inhibitors displaying 2-anilinopyrimidine partial structures [14].

The Aurora kinases A, B and C are serine/threonine kinases involved in the regulation of the cell division process [11]. Although sharing structural similarity, they vary in cellular and subcellular localization. Thus, Aurora B is expressed in virtually all cells, while Aurora C is mainly expressed in germ cells. Aurora A is involved in the transition from the G2 to the M phase of the cell cycle by promoting centrosome maturation and mitotic spindle development. Aurora B and Aurora C are involved in the binding of chromosomes to kinetochores and in the segregation of chromosomes. All Aurora kinases appear to act as oncogenes by promoting cell proliferation and cell survival [12]. Dysfunction or overexpression of Aurora kinases has been observed in various human tumors, such as breast, lung, ovarian, prostate and colon cancer [13,14]. Consequently, in recent years a number of Aurora kinase inhibitors have been developed as anticancer drugs, of which several were evaluated in clinical trials [14,15,16,17]. The depicted structures PF-03814735 (**3**, IC_50_ Aurora A = 0.8 nM), CYC116 (**4**, K_i_ Aurora A = 8 nM), MLN8054 (**5**, IC_50_ Aurora A = 4 nM) and alisertib (**6**, also known as MLN8237, IC_50_ Aurora A = 1.2 nM) [14] display 2-anilinopyrimidine elements and in this respect share a certain degree of structural similarity with members of the new compound class 2 (Figure 2). Alisertib is probably the most advanced Aurora kinase inhibitor in clinical studies. However, in a randomized phase III trial in patients with relapsed and refractory peripheral T-cell lymphoma (PTCL), alisertib has not been significantly superior over the comparator drug regimens (pralatrexate, romidepsin or gemcitabine, respectively) [18]. Recently it has been shown that alisertib selectively kills HPV-positive cervical cancer cells, and for a targeted application an intravaginal alisertib-loaded ring was constructed [19]. Based on this, a developing research area on Aurora kinases as therapeutic targets and new inhibitor chemotypes are of interest.

With the aim to generate structure–activity relationship information, a small collection of congeners of prototype **2a** was synthesized. Based on the results of crystal structure analysis and on corresponding docking experiments, we identified the aniline as a promising area for molecular modification. Besides small customary substituents that were added for decoration of the aniline ring, we also introduced sulfamoylaminoalkoxy groups, which were envisaged to modify the structures without decreasing the biological activity and without increasing the lipophilicity. Evaluation of representatives **2e** and **2n** of the new protein kinase inhibitor class in the cancer cell line panel of the National Cancer Institute (NCI) revealed considerable antiproliferative activity on tumor cell lines in the single digit micromolar concentration range.

## 2. Results

### 2.1. Chemistry

**Scheme 1:**
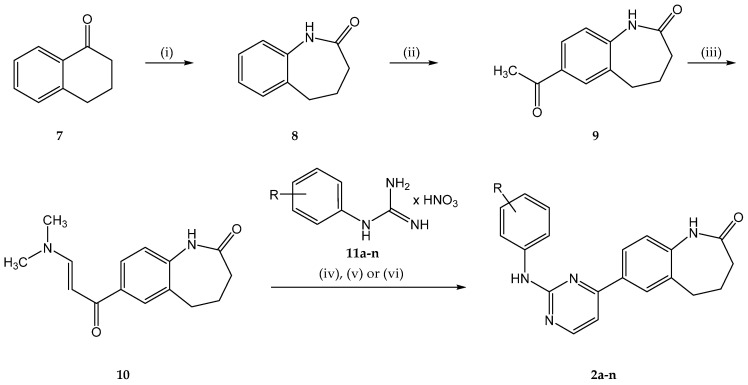
Anilinopyrimidines **2a**–**n**. Reagents and conditions: (i) NaN_3_, H_2_SO_4_, AcOH, rt → 60 °C, 30 min, 67% [20]; (ii) AcCl, AlCl_3_, CS_2_, reflux, 1 h, then rt, 3 h, 67% [21]; (iii) DMF-DMA, 110–115 °C, 13 h, 59% [22]; (iv) propan-2-ol, NaOH, reflux, 15–30 h, (v) propan-2-ol, NaOH, microwave irradiation: 150 W, 140 °C, 30–90 min or (vi) DMF, NaOH, microwave irradiation: 150 W, 175 °C, 30–90 min. For substituents R refer to Table 3.

The title compounds were prepared starting from α-tetralone (7), which was reacted with hydrazoic acid to yield 1-benzazepine-2-one 8 by means of a Schmidt reaction [20]. Subsequent Friedel-Crafts acylation with acetyl chloride in carbon disulfide led to the acetophenone derivative 9 [21]. The enaminone 10 was then prepared from 9 by prolonged heating with dimethylformamide dimethyl acetal [22]. Eventually, heating **10** with appropriate *N*-arylguanidinium nitrates **11a–n** under conventional [23] or microwave conditions furnished the anilinopyrimidines **2a**–**n** (Scheme 1).

**Scheme 2:**
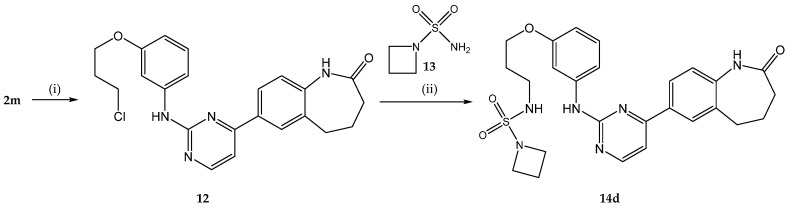
Preparation of sulfamoylaminoalkoxy-substituted compounds **14a**–**g** exemplified by the synthesis of **14d**. Reagents and conditions: (i) 1-bromo-3-chloropropane, Cs_2_CO_3_, ACN, reflux, 2 h, 25% and (ii) K_2_CO_3_, NaI, DMF, 80 °C, 22 h, 34%.

For the attachment of larger substituents with sulfamide substructures, the phenols **2m** and **2n** were reacted with 1-bromo-3-chloropropane in the presence of cesium carbonate by means of a Williamson synthesis. The resulting chloroalkane **12** was then reacted with a suitable sulfamide in the presence of a base (potassium carbonate or sodium hydride, respectively) to yield the sulfamoylaminoalkoxylated derivatives of series **14**. The procedure is outlined for the example **14d** in Scheme 2.

### 2.2. Molecular Docking

**Figure 3:**
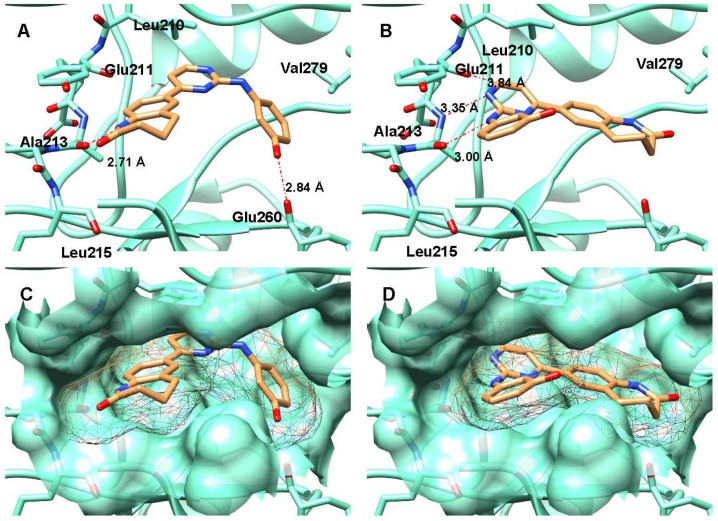
Two different binding modes of **2m** (sand brown) in the ATP binding pocket of aurora kinase A (PDB: 2WTV, aquamarine [24]) predicted by docking experiments. Hydrogen bonds are shown in red chain lines. Left (**A**,**C**): Without application of constraints; right (**B**,**D**): constraints were defined between the nitrogen atom of the aniline partial structure of **2m** and the carbonyl group of Ala213 (gk+3). (**A**): The NH of the lactam ring interacted with Ala213 (gk+3) via a hydrogen bond (D–A = 2.71 Å). In addition, a hydrogen bond (D–A = 2.84 Å) between the hydroxy group of **2m** and Glu260 was predicted. (**B**): The hydrogen bond defined as constraint (D–A = 3.00 Å) was observed. Two additional hydrogen bonds were formed between the hinge region (Ala213 and Glu211) and the pyrimidine ring of **2m**, involving the C(6)H group as a donor and ring nitrogen 1 as an acceptor. (**C**): Surface of the ATP-binding pocket and the ligand without constraints. The anilinopyrimidine partial structure fills the phosphate binding region. (**D**): Surface representation of the binding pocket with defined constraint. The hydroxy group was directed towards the solvent, while the benzazepine-2-one partial structure filled the hydrophobic region and the phosphate binding region.

For a structure-based design of congeners of the prototype **2a**, insight into the orientation mode of the structures as ligands in the Aurora A protein was required. It was assumed that the molecules would bind in the ATP binding pocket via their anilinopyrimidine substructure towards the hinge region, similar to the binding mode of MLN8054 (**5**) displayed in the corresponding cocrystal structure with Aurora-A (PDB: 2WTV) [24]. Docking experiments using GOLD [25] and the mentioned crystal structure however yielded poses that appeared unlikely, since the anilinopyrimidine structure was not addressing the hinge area (Figure 3A,C). Therefore, we applied a constraint for a hydrogen bond between Ala213 and the NH-group of the aniline moiety of the ligands, as exemplified for congener **2m** in Figure 3B,D.

Employing the constraint, the docking procedure indeed placed the prototype **2a** and its congeners in the ATP binding pocket similar to the orientation of MLN8054 (**5**) in the original crystal structure (PDB: 2WTV) [24] as depicted for **2m** in Figure 3B,D. Direct interactions were predicted with three hydrogen bonds between the ligand **2m** and Ala213 and Glu211 of the hinge region of Aurora A. As described below, the crystal structures of congeners **2a** and **2c** later corroborated the general binding orientation predicted under this constraint.

### 2.3. Crystal Structure Analyses

**Figure 4:**
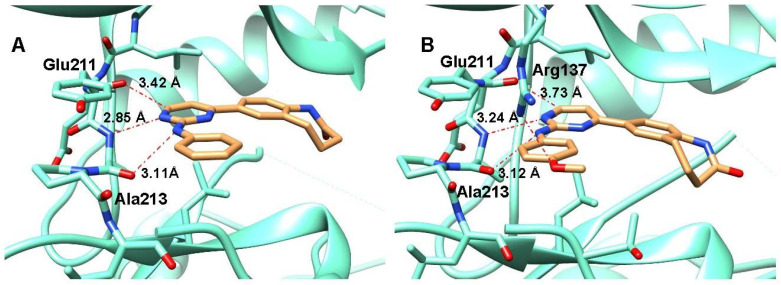
Cocrystal structures of Aurora A in complex with **2a** (**A**) and **2c** (**B**). (**A**): The characteristic donor–acceptor–donor triad of hydrogen bonds from the inhibitors to the amino acids Glu211 and Ala213 of the hinge area is observed. (**B**): In the complex with **2c**, an additional weak hydrogen bond between the methoxy group and Arg137 (D–A = 3.26 Å) is formed. Hydrogen bonds are shown in red chain lines.

To confirm the binding mode of the members of the new Aurora A inhibitor class, we determined the crystal structures of the kinase in complex with the representative inhibitors **2a** and **2c** (Table 1). The two cocrystallized inhibitors shared similar binding modes, which were in agreement with the ones suggested by the docking analyses employing the constraint between the aniline NH and Ala213 of the protein (Figure 4). While the anilinopyrimidine elements of the inhibitors adopted flat configurations in the ATP adenine binding pocket, the benzazepine-2-one partial structures filled the hydrophobic region. Towards the hinge area, the pyrimidine ring formed a weak hydrogen bond from C(6)H to Glu211. Another hydrogen bond was detected between the carbonyl group of Ala213 and the NH group of the aniline element. The third contact was established between the pyrimidine nitrogen atom 1 and the NH group of Ala213. In the structure with **2c,** an additional interaction between the methoxy group of the ligand and Arg137 was observed.

The main result drawn from the docking studies and the crystal structure analyses was the finding that the 3- and 4-positions of the aniline ring are oriented towards the entrance of the ATP binding pocket. Therefore, these positions were assumed as suitable attachment points for further substituents that would not interfere unfavorably with binding.

### 2.4. Protein Kinase Inhibition

The inhibitory activity of prototype **2a** was determined in a panel of 24 tumor-relevant protein kinases using a radiometric assay. Only the kinases Aurora A, VEGF-R2 and VEGF-R3 were inhibited with single digit micromolar IC_50_ values (Table 2). Structural modifications were then implemented, particularly directed to improve inhibition of Aurora A. The so-designed congeners with small substituents at the aniline ring **2b**–**2n** were examined for inhibition of Aurora A and of VEGF-R2, VEGF-R3 and Aurora B for comparison (Table 3). For the group of analogues with sulfamoylaminoalkoxy substituents **14** the results for inhibition of Aurora A are displayed in Table 4.

### 2.5. Kinetic Solubility

Solubility in aqueous media is of utmost importance for orally administered drugs, as it significantly influences absorption from the gastrointestinal tract. Poorly soluble drugs are hardly suitable as development candidates, since in the course of drug development solubility often decreases because of molecule enlargement [28,29,30]. As a rule of thumb, active ingredients with moderate permeability and potency should display a solubility of at least 60 µg/mL [31], which corresponds to a concentration of 150 µM at an average molecular weight of 400. Since the unsubstituted prototype **2a** showed a significantly lower solubility then this benchmark value, the modification of the prototype either involved the addition of only very small additional substituents to the aniline ring (compounds **2b**–**2n**) or of sulfamoylaminoalkyloxy elements (compounds **14a**–**g**). Although increasing molecular weight, the latter substituents contribute additional sp^3^-hybridized carbon atoms [32] and the polar sulfamide group and therefore were expected to cause at least no solubility impairment. Table 4 shows the kinetic solubility of the representatives of series **14**, determined by nephelometry [33].

### 2.6. Antiproliferative Activity on Cancer Cell Lines

The new dual Aurora A/VEGF-R inhibitors **2e** and **2n** were selected by the National Cancer Institute for screening in its broad cancer cell line panel [34,35,36]. The NCI in vitro assay is performed by incubation of cells with test compounds for 48 h and subsequent cell fixation with trichloroacetic acid. The pink dye sulforhodamin B is added, which binds to the proteins of the fixed cells. After washing and solubilizing the bound stain, the optical density is measured spectrophotometrically to determine the relative proliferation of treated and untreated cells [37,38]. Results of the assays with **2e** and **2n** are shown in Table 5.

## 3. Discussion

Although significant progress in tumor therapy was achieved in the last two decades with numerous protein kinase inhibitors, relatively few of the tumor-relevant protein kinases have been exploited as targets in clinical therapy by selective inhibitors. New, alternative basic structures are of interest for the development of appropriate drugs acting on these previously unaddressed protein kinases [1,2]. We assumed that structural elements of known protein kinase inhibitors are particularly well suited as binding motifs for complementary regions in the ATP binding pocket of kinases and that new chemotypes for protein kinase inhibitors could be identified by recombining such partial structures. We have termed the corresponding procedure “cut and glue” method and applied it to the already published compound class of dual PLK1/VEGF-R2 inhibitors **1**. Formal decomposition of this initial structure on the annulation side between the azepinone ring and the pyrimidine ring and relinking between positions 7 and 4 of these cycles resulted in the new basic structure **2** (Figure 1). Initially, this approach did not target any specific structure of a particular protein kinase. Instead, the prototype **2a** was screened for inhibition of 24 tumor relevant protein kinases by assessment of IC_50_ values with a radiometric assay (Table 2).

The results show that **2a** inhibited three tumor relevant protein kinases in the single-digit micromolar concentration range, namely Aurora A, VEGF-R2 and VEGF-R3, albeit with IC_50_ values being at least three orders of magnitude higher than displayed by the established inhibitors mentioned in the introduction. The inhibition of the VEGF receptor kinases was not very surprising, since the initial structure **1** also inhibits these targets [4]. In contrast, no inhibition of PLK1 was observed in **2a** in contrast to **1**. Instead, an inhibition of the Aurora A kinase by **2a** was identified. For comparison, the 2-anilinopyrimidine substructure is the common characteristic structural unit of well-known Aurora A kinase inhibitors such as PF-03814735 (**3**) [41], CYC116 (**4**) [42], MLN8054 (**5**) [43] and alisertib (**6**) [44]. A publicly accessible X-ray structure of a complex of the inhibitor MLN8054 and Aurora A shows that the inhibitor in the ATP binding pocket is bound to the amino acids of the hinge region via the 2-anilinopyrimidine [24]. Our docking analyses with structural class **2** inhibitors could not reproduce this binding mode at first, but instead a binding type was obtained in which the lactam structure of the seven-membered ring addressed the hinge region as shown for **2m** in Figure 3A,C. Despite a good filling of the binding pocket by the inhibitor, we considered this orientation improbable because it only forms a single hydrogen bridge to the hinge region.

Therefore, we crystallized Aurora A with the characteristic representatives **2a** and **2c**, whose structures were elucidated by X-ray analysis. These structures showed high analogy to the binding of MLN8054 to the protein and met our expectation that the 2-anilinopyrimidine structure is oriented towards the hinge region, where a triad of hydrogen bonds to the amino acids Glu211 and Ala213 is formed (Figure 3). By setting a constraint between Ala 213 and the aniline-NH in the docking experiments, corresponding poses for newly designed analog structures were generated, as shown in Figure 3B,D. Based on this orientation, substituents were attached to positions 3 and 4 of the aniline substructure of **2a** for optimization purposes. The results of the tests presented in Table 3 show that small substituents in position 4 (halogen, hydroxy and alkoxy) only slightly change the inhibitory activity on Aurora A and B as well as on VEGF-R2 and VEGF-R3. In contrast, a nitro group in position 4 (**2i**) causes the complete loss of aurora inhibition and also leads to a significant reduction in activity at the VEGF receptor kinases. Similarly, substituents in the 2-position (**2j**–**2l)** are detrimental for kinase inhibition, presumably due to twisting of the aniline ring from its original plane for steric reasons.

For further optimization, **2a** was decorated with larger substituents in position 3 of the aniline ring, which is facing the solvent according to the X-ray structure. Through this modification we intended to generate additional contacts with the protein kinase near the entrance area of the ATP binding pocket. Enlargement of drug molecules, however, often results in increased lipophilicity and in decreased aqueous solubility. Poor solubility is often observed in protein kinase inhibitors and represents a major problem in preclinical drug development [45,46]. An orally administered drug of medium potency and medium permeability should have a solubility of at least 60 µg/mL [31], which corresponds to a concentration of 150 µM at a relative molar mass of 400.

**Figure 5:**
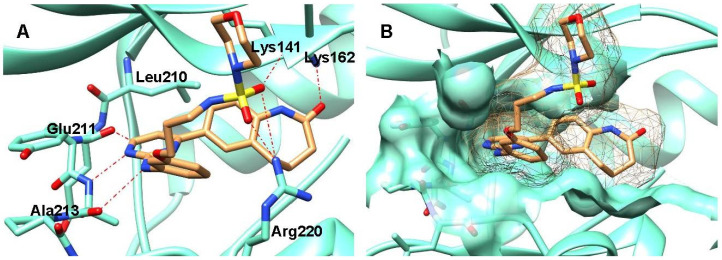
Docking result of **14b** in the ATP binding pocket of Aurora A Kinase (PDB 7AYH). (**A**): Hydrogen bonds are formed between Glu211 and the CH group of the pyrimidine ring (D–A = 2.59 Å), between the aniline NH group and Ala213 (D–A = 3.17 Å) and between the pyrimidine nitrogen atom and Ala213 (D–A = 2.54 Å). There is also a hydrogen bond between the carbonyl group of benzazepin-2-one and Lys162. The sulfonyl group interacts with the amino acids Lys141 and Arg220. (**B**): Surface representation of the ATP-binding pocket and ligand **14b**.

For the compounds presented here, the kinetic solubility (solubility of the fastest precipitating form) was assessed. Since this property can be determined relatively quickly, it is suitable for comparative considerations in a number of similar compounds. Displaying a kinetic solubility of about 50 µM, even the unsubstituted basic structure **2a** was significantly less soluble than the target value. While the introduction of phenolic hydroxyl groups increased the solubility (e.g., **2m**, **n**, Table 4), phenols are prone to rapid metabolic turnover, which is considered a disadvantage in drug design. We therefore introduced sulfamide structures into the 3-position, which were bound to the aniline ring via a hydrocarbon chain and an ether oxygen atom (compounds **14a**–**g**). The sulfamide structure should form additional contacts in the entrance area of the ATP binding pocket and limit the lipophilicity of the substances by its polarity. The additional sp^3^-hybridized carbon atoms of the linker chain should also have a positive effect on solubility [32]. Docking experiments with the compounds **14a**–**14g** designed in this way resulted in useful poses, which were characterized by additional contacts in the form of hydrogen bonds between ligand and protein. Figure 5 shows the result of a docking experiment with the sulfamide-substituted compound **14b** in the protein structure taken from PDB 7AYH. In this orientation, the morpholinosulfamoylpropoxy chain protrudes from the ATP binding pocket into the solvent as desired, but can still form a pair of hydrogen bonds to the guanidino group of Arg220 in the entry region. In this pose, the inhibitor is also pushed into the binding pocket so far that a hydrogen bridge is formed between the carbonyl carbon atom of the lactam group and Lys162.

The results listed in Table 4 only partially met our expectations with the representatives of the **14** series. Although the compounds exhibited inhibitory activity on the Aurora A kinase in the same order of magnitude as the most potent Series **2** representatives, a significant improvement over the unsubstituted basic structure **2a** was not achieved. In addition, a slight decrease in solubility was observed compared to the unsubstituted structure **2a**. In view of these results, the inhibition of VEGF receptor kinases was not further investigated for the congener series **14**.

Simultaneous inhibition of the Aurora A kinase and the VEGF-R2/3 receptor kinases may represent an interesting dual activity for the treatment of cancer. The National Cancer Institute selected the compounds **2e** and **2n** as representatives with particularly high inhibitory activity on the above kinases for testing in the IVCLS (in vitro cell line screening). In this screening, compounds are tested for antiproliferative activity on about 60 in vitro cultivated tumor cell lines [34,35,36,47]. Based on this very broad screening on cell lines from nine tumor entities the general antiproliferative potency of compounds is determined. In addition, the bioinformatic tool COMPARE allowed one to draw conclusions on the molecular target of the compounds from the activity patterns on the different cell lines [47,48,49,50,51]. Similar molecular mechanisms of action of two compounds are assumed if a Pearson correlation coefficient (PCC) of > 0.6 is calculated by COMPARE [52,53]. Table 5 shows the GI_50_ values for test compounds **2e** and **2n**. For the majority of the cell lines tested, both compounds caused growth inhibition in the single-digit micromolar concentration range. Only the cell line MDA-MB-231/ATCC showed high resistance to both test compounds with GI_50_ values >100 µM. The selectivity profile of the two compounds is not very pronounced given the overall very similar proliferation inhibition values across all cell lines. A COMPARE analysis determined a PCC = 0.61 for the patterns of test compounds **2e** and **2n**, which is consistent with an analogous mechanism of action. However, whether this mechanism is actually based on the inhibition of the inhibited Aurora kinases and VEGF receptor kinases cannot be deduced from these profiles.

The new compound class **2** is an example of how the “cut and glue” technique is used to discover new chemotypes for protein kinase inhibitors with unexpected selectivity profiles. However, established Aurora A kinase inhibitors such as the compounds **3**–**6** presented in Figure 2 with IC_50_ values in the single-digit nanomolar range are far more potent than the representatives of the new compound class **2**. The results presented here indicate that a structural modification merely by substitution at the aniline ring of **2a** is not sufficient for a significant optimization. Instead, in future studies modifications should be made to the benzazepinone element of **2** based on the structural information presented here. Such further studies appear to be worthwhile in view of the strong antiproliferative properties of **2e** and **2n**. In further studies, based on the “cut and glue” technique, the benzazepinone and anilinopyrimidine scaffolds can also be linked via other positions. Such alternative combinations open pathways to multiple new chemotypes that are also expected to display biological activity.

## 4. Materials and Methods

### 4.1. General Information

The starting materials and reagents were purchased from Acros Organics (Geel, Belgium), Alfa Aesar (Karlsruhe, Germany) and Sigma-Aldrich (Steinheim, Germany). All purchased reagents and solvents were used without further purification. Silica gel 60 Å was used for purification by column chromatography. Reaction monitoring was performed using thin layer chromatography (TLC): Polygram SIL G/UV254, 0.2 mm silica gel 60, 40 mm × 80 mm (Macherey-Nagel, Düren, Germany), visualization by UV light (254 and 366 nm). The melting points (m.p.) were detected in open-glass capillaries on an electric variable heater (Barnstead Electrothermal IA 9100, Southend-on-Sea, Essex, UK). The infrared spectra were recorded on a Thermo Nicolet FT-IR 200 spectrometer (Thermo Nicolet, Madison, WI, USA) using KBr pellets. ^1^H-NMR, ^13^C-NMR and ^13^C-DEPT135 spectra were recorded on Bruker Avance DRX-400, Bruker Avance III 400, Bruker Avance II 600 or Bruker Avance III HD 500 spectrometers (Bruker Biospin, Rheinstetten, Germany) (at the NMR laboratories of the Chemical Institutes of the Technische Universität Braunschweig) in *d*_6_-DMSO with tetramethylsilane as an internal standard (δ = 0 ppm). Chemical shifts are presented as parts per million (ppm) in relation to internal standard. Atmospheric pressure chemical ionization (APCI) spectra were determined with an expression^L^ CMS spectrometer (Advion, Ltd., Harlow, UK); the APCI source was coupled with ASAP (atmospheric solids analysis probe) and the measurements were performed in the positive mode. The elemental analyses were performed on a CE Instruments Flash EA^®^ 1112 Elemental Analyzer (Thermo Quest, San Jose, CA, USA). Purity of test compounds was determined by high performance liquid chromatography (Merck Hitachi Elite LaChrom system; Hitachi High Technologies Corporation, Tokyo, Japan) diode array detector: L-2450; pump: L-2130; autosampler: L-2200; organizer box: L-2000; column: Merck LiChroCART 125-4, LiChrospher 100 RP-18 (5 μm) (Merck, Darmstadt, Germany); flow rate: 1.000 mL/min; detection wavelength: 254 nm and 280 nm (isocratic); overall run time: 15 min (isocratic); AUC, 100% method; t_s_ = dead time related to DMSO; t_ms_ = retention time. For isocratic elution different acetonitrile/water mixtures were used. Microwave-assisted syntheses were carried out in a CEM focused microwave^TM^ synthesis system, type Discover; ChemDriver^TM^ application software program; reaction vessel: 10 mL with Teflon septum (closed system, CEM GmbH, Kamp-Lintfort, Germany), equipped with a Jun-Air compressor (Blue Line Model, Jun-Air International, Nørresundby, Denmark). Preparative HPLC: LaPrep P 110 HPLC pump; LaPrep P 311 spectral photometer; LaPrep P 216 fraction collector; injection loop 5 mL; software: EZChrom Elite, version 3.1.4.; column 125 mm LiChrospher 100 RP-18, 12 µM (Merck, Darmstadt, Germany); detection wavelength 245 nm, flow rate 40 mL/min, run time 20–60 min. For preperative HPLC, samples of 20–60 mg were dissolved in a mixture (4 mL) of DMSO and the indicated eluent using a sandwich injection procedure between two DMSO layers (0.5 mL each).

### 4.2. Syntheses

#### 4.2.1. Syntheses of Intermediates

Synthesis procedures and analytical characterization of enaminone **10**, *N*-arylguanidinium nitrates **11a**–**n**, and *N*-alkylated sulfamides (**13** and its analogues) are presented in the Supplementary Materials.

#### 4.2.2. General Procedure A for the Synthesis of 7-(2-Anilinopyrimidin-4-yl)-1,3,4,5-tetrahydro-2*H*-1-benzazepin-2-ones **2b,d,e,f,g,h,i,k,l**

7-[3-(Dimethylamino)acryloyl]-1,3,4,5-tetrahydro-2*H*-benzo[*b*]azepin-2-one (**10**) (1 eqivalent), an appropriate *N*-arylguanidinium nitrate (1–1.2 equivalents) and sodium hydroxide (1–2 equivalents) are dissolved in DMF (2–4 mL) and heated in a 10 mL microwave vessel at 175 °C by using a microwave reactor (150 Watt, maximum pressure: 290 psi). After cooling to room temperature, a precipitate is formed, which is collected by filtration, washed with water and purified by recrystallization.

#### 4.2.3. General Procedure B for the Synthesis of 7-(2-Anilinopyrimidin-4-yl)-1,3,4,5-tetrahydro-2*H*-1-benzazepin-2-ones **2c,m,n**

7-[3-(Dimethylamino)acryloyl]-1,3,4,5-tetrahydro-2*H*-benzo[*b*]azepin-2-one (**10**) (1 eqivalent), an appropriate *N*-arylguanidinium nitrate (1–1.2 equivalents) and sodium hydroxide (1–2 equivalents) are dissolved in propan-2-ol (2–4 mL) and heated at 140 °C in a 10 mL microwave vessel using a microwave reactor (150 Watt, maximum pressure: 290 psi). After cooling to room temperature, a precipitate is formed, which is collected by filtration, washed with water and purified by recrystallization.

#### 4.2.4. General Procedure C for the Synthesis of 2-Methyl-1,2,5-thiadiazolidine-1,1-dioxide Derivatives **14a,e**

2-Methyl-1,2,5-thiadiazolidine-1,1-dioxide (**13a**) (1 equivalent) is dissolved in DMF (9–30 mL). Sodium hydride (60% dispersion in mineral oil, 1.2–1.6 equivalents) is gradually added to the solution at 0 °C. Subsequently the mixture is stirred for 1 h at room temperature under N_2_-atmosphere. Then the appropriate haloalkane (0.03–1.3 equivalents) is added in portions to the mixture at 0 °C. After stirring for 15–48 h at room temperature, the mixture is quenched with water (10 mL) and extracted with ethyl acetate (3 × 30 mL). The combined organic phases are washed with saturated sodium chloride solution (30 mL), dried with sodium sulfate and evaporated to dryness. The residue is purified by column chromatography and/or crystallization.

#### 4.2.5. General Procedure D for the Synthesis of *N*-Alkylated Sulfamide Derivatives **14b,c,d,f,g**

The appropriate haloalkane (1 equivalent) is dissolved in DMF (9 mL). Successively potassium carbonate (4–5 equivalents), *N*-alkylated sulfamide **14b–d** (4–5 equivalents), and sodium iodide (0.6 equivalents) are added to the suspension and the mixture is heated for 22–51 h at 80 °C. After cooling to room temperature, water (9 mL) is added and the mixture is extracted with ethyl acetate (3 × 20 mL). The combined organic phases are washed with water (2 × 50 mL), dried with sodium sulfate and evaporated to dryness. The residue is purified by preparative HPLC.

#### 4.2.6. Detailed Chemical Synthesis Procedures and Characterization

*7-(2-Anilinopyrimidin-4-yl)-1,3,4,5-tetrahydro-2H-1-benzazepin-2-one (**2a**)*: 7-[3-(Dimethylamino)acryloyl]-1,3,4,5-tetrahydro-2*H*-benzo[*b*]azepin-2-one (**10**, 77 mg, 0.33 mmol), *N*-arylguanidinium nitrate (**11a**, 65 mg, 0.33 mmol) and sodium hydroxide (13 mg, 0.33 mmol) in 4 mL DMF were heated at reflux for 16.5 h. After cooling to room temperature, the mixture was poured on water (50 mL). A precipitate was formed, which was collected by filtration, washed with water and purified by crystallization from ethanol to yield colorless crystals (36 mg, 36%). m.p.: 249 °C; IR (KBr): ῦ _max_ 3247 (NH), 3183 (NH), 1668 cm^−1^ (C=O); ^1^H-NMR (*d_6_*-DMSO, 400 MHz): δ (ppm) = 2.13–2.23 (m, 4H, CH_2_-CH_2_), 2.78–2.81 (m, 2H, CH_2_), 6.96 („t”, *J* = 7.3 Hz, 1H, CH_2_), 7.12 (d, *J* = 5.3 Hz, 1H, ArH), 7.32 (t, *J* = 7.6 Hz, 2H, ArH), 7.38 (d, *J* = 5.3 Hz, 1H, pyrimidine-H), 7.84 (m, 2H, ArH), 8.04 (dd, *J* = 8.2, 2.0 Hz, 1H, ArH), 8.09 (d, *J* = 1.7 Hz, 1H, ArH), 8.52 (d, *J* = 5.2 Hz, 1H, pyrimidine-H), 9.63 (s, 1H, NH), 9.77 (s, 1H, NH); ^13^C-NMR (*d_6_*-DMSO, 101 MHz): δ (ppm) = 27.8, 30.3, 33.1 (CH_2_), 107.5, 118.8 (2C), 121.3, 121.6, 125.9, 128.4 (2C), 128.5, 158.8 (CH), 132.6, 133.7, 140.6, 141.5, 160.1, 163.1, 173.3 (C); C_20_H_18_N_4_O (330.38): calc. C 72.71, H 5.49 N 16.96, found C 72.41, H 5.53, N 16.65; APCI-MS: *m/z* (%) 331 [M+H]^+^ (100); HPLC (isocr.): 99.1% at 254 nm, 99.8% at 280 nm, t_ms_ = 2.63 min, t_m_ (DMSO) = 1.02 min (ACN/H_2_O 50:50).

*7-[2-(4-Toluidino)pyrimidin-4-yl]-1,3,4,5-tetrahydro-2H-1-benzazepin-2-one (**2b**):* Synthesis according to General Procedure A with 7-[3-(dimethylamino)acryloyl]-1,3,4,5-tetrahydro-2*H*-benzo[*b*]azepin-2-one (**10**, 258 mg, 1.00 mmol) and *N*-(4-methylphenyl)guanidinium nitrate (**11b**, 233 mg, 1.10 mmol), reaction time 2.5 h. Recrystallization from ethyl acetate gave a yellow powder (66 mg, 19%). m.p.: 223 °C; IR (KBr): ῦ _max_ 3282 (NH), 3195 (NH), 1668 cm^−1^ (C=O); ^1^H-NMR (*d_6_*-DMSO, 400 MHz): δ (ppm) = 2.15–2.25 (m, 4H, CH_2_-CH_2_), 2.27 (s, 3H, CH_3_), 2.77–2.81 (m, 2H, CH_2_), 7.10–7.13 (m, 3H, ArH), 7.34 (d, *J* = 5.3 Hz, 1H, pyrimidine-H), 7.70–7.72 (m, 2H, ArH), 8.03 (dd, *J* = 8.1, 2.0 Hz, 1H, ArH), 8.07 (d, *J* = 2.0 Hz, 1H, ArH), 8.49 (d, *J* = 5.1 Hz, 1H, pyrimidine-H), 9.50 (s, 1H, NH), 9.74 (s, 1H, NH); ^13^C-NMR (d_6_-DMSO, 126 MHz): δ (ppm) = 20.3 (CH_3_), 27.7, 30.2, 33.1 (CH_2_), 107.2, 118.8 (2C), 121.6, 125.8, 128.3, 128.8 (2C), 158.7 (CH), 130.0, 132.6, 133.7, 138.0, 141.4, 160.1, 163.0, 173.2 (C); C_21_H_20_N_4_O (344.41): calc. C 73.23, H 5.85 N 16.27, found C 73.18, H 5.85, N 15.69; APCI-MS: *m/z* (%) 345 [M+H]^+^ (100); HPLC (isocr.): 98.9% at 254 nm, 99.5% at 280 nm, t_ms_ = 2.86 min, t_m_ (DMSO) = 1.03 min (ACN/H_2_O 60:40).

*7-[2-(4-Methoxyanilino)pyrimidin-4-yl]-1,3,4,5-tetrahydro-2H-1-benzazepin-2-one (**2c**)*: Synthesis according to General Procedure B with 7-[3-(dimethylamino)acryloyl]-1,3,4,5-tetrahydro-2*H*-benzo[*b*]azepin-2-one (**10**, 103 mg, 0.4 mmol) and *N*-(4-methoxyphenyl)guanidinium nitrate (**11c**, 110 mg, 0.480 mmol), reaction time 30 min. Recrystallization from ethanol 70% gave ochre-colored crystals (82 mg, 57%). m.p.: 195 °C; IR (KBr): ῦ _max_ 3276 (NH), 3189 (NH), 1670 cm^−1^ (C=O); ^1^H-NMR (*d*_6_-DMSO, 400 MHz): δ (ppm) = 2.15–2.22 (m, 4H, CH_2_-CH_2_), 2.77–2.81 (m, 2H, CH_2_), 3.74 (s, 3H, OCH_3_), 6.90–6.93 (m, 2H, ArH), 7.10 (d, *J* = 8.3 Hz, 1H, ArH), 7.31 (d, *J* = 5.3 Hz, 1H, pyrimidine-H), 7.69–7.73 (m, 2H, ArH), 8.02 (dd, *J* = 8.3 Hz, *J* = 2.0 Hz, 1H, ArH), 8.06 (d, *J* = 2.0 Hz, 1H, ArH), 8.47 (d, *J* = 5.3 Hz, 1H, pyrimidine-H), 9.43 (s, 1H, NH), 9.75 (s, 1H, NH); ^13^C-NMR (*d*_6_-DMSO, 126 MHz): δ (ppm) = 55.1 (CH_3_), 27.7, 30.2, 33.1 (CH_2_), 106.2, 113.7 (2C), 120.5 (2C), 121.6, 125.8, 128.3, 158.7 (CH), 132.6, 133.6, 133.7, 141.4, 154.7, 160.2, 163.0, 173.2 (C); C_21_H_20_N_4_O_2_ (360.41): calc. C 69.98, H 5.59 N 15.55, found C 69.78, H 5.66, N 15.05; APCI-MS *m/z* (%) 361.2 [M+H]^+^ (100), 375.2 [M+15]^+^ (30); HPLC (isocr.): 99.7% at 254 nm, 99.8% at 280 nm, t_ms_ = 2.62 min, t_m_ (DMSO) = 1.03 min (ACN/H_2_O 60:40).

*7-[2-(4-Ethoxyanilino)pyrimidin-4-yl]-1,3,4,5-tetrahydro-2H-1-benzazepin-2-one (**2d**):* Synthesis according to General Procedure A with 7-[3-(dimethylamino)acryloyl]-1,3,4,5-tetrahydro-2*H*-benzo[*b*]azepin-2-one (**10**, 266 mg, 1.00 mmol) and *N*-(4-ethoxyphenyl)guanidinium nitrate (**11d**, 258 mg, 1.10 mmol), reaction time1 h. Recrystallization from ethanol gave violet crystals (170 mg, 45%). m.p.: 205 °C; IR (KBr): ῦ _max_ 3278 (NH), 3181 (NH), 1671 cm^−1^ (C=O); ^1^H-NMR (d_6_-DMSO, 400 MHz): δ (ppm) = 1.32 (t, *J* = 7.1 Hz, 3H, CH_3_), 2.10–2.24 (m, 4H, CH_2_-CH_2_), 2.77–2.81 (m, 2H, CH_2_), 3.99 (q, *J* = 7.1 Hz, 2H, CH_2_ (OCH_2_CH_3_)), 6.88–6.92 (m, 2H, ArH), 7.10 (d, *J* = 8.3 Hz, 1H, ArH), 7.31 (d, *J* = 5.3 Hz, 1H, pyrimidine-H), 7.67–7.72 (m, 2H, ArH), 8.02 (dd, *J* = 8.3, 2.0 Hz, 1H, ArH), 8.06 (d, *J* = 2.0 Hz, 1H, ArH), 8.46 (d, *J* = 5.3 Hz, 1H, pyrimidine-H), 9.42 (s, 1H, NH), 9.75 (s, 1H, NH); ^13^C-NMR (*d_6_*-DMSO, 101 MHz): δ (ppm) = 14.7 (CH_3_), 27.8, 30.2, 33.1, 63.1 (CH_2_), 106.9, 114.3 (2C), 120.6 (2C), 121.6, 125.8, 128.3, 158.9 (CH), 132.7, 133.6, 133.7, 141.4, 153.4, 160.2, 163.0, 173.2 (C); C_22_H_22_N_4_O_2_ (374.44): calc. C 70.57, H 5.92 N 14.96, found C 70.44, H 5.99, N 14.68; APCI-MS *m/z* (%) 375.2 [M+H]^+^ (100); HPLC (isocr.): 99.3% at 254 nm, 99.6% at 280 nm, t_ms_ = 3.71 min, t_m_ (DMSO) = 1.02 min (ACN/H_2_O 50:50).

*7-[2-(4-Hydroxyanilino)pyrimidin-4-yl]-1,3,4,5-tetrahydro-2H-1-benzazepin-2-one (**2e**)*: Synthesis according to General Procedure A with 7-[3-(dimethylamino)acryloyl]-1,3,4,5-tetrahydro-2*H*-benzo[*b*]azepin-2-one (**10**, 103 mg, 0.40 mmol) and *N*-(4-hydroxyphenyl)guanidinium nitrate (**11e**, 103 mg, 0.480 mmol), reaction time 1 h. Recrystallization from ethanol 70% gave ochre-colored crystals (60 mg, 43%). m.p.: 291 °C; IR (KBr) ῦ _max_ 3251 (NH), 3196 (NH), 1653 cm^−1^ (C=O); ^1^H-NMR (*d*_6_-DMSO, 400 MHz): δ (ppm) = 2.15–2.22 (m, 4H, CH_2_-CH_2_), 2.76–2.80 (m, 2H, CH_2_), 6.71–6.75 (m, 2H, ArH), 7.09 (d, *J* = 8.3 Hz, 1H, ArH), 7.27 (d, *J* = 5.3 Hz, 1H, pyrimidine-H), 7.53–7.57 (m, 2H, ArH), 8.00 (dd, *J* = 8.2, 2.1 Hz, 1H, ArH), 8.05 (d, *J* = 1.9 Hz, 1H, ArH), 8.44 (d, *J* = 5.2 Hz, 1H, pyrimidine-H), 9.03 (s, 1H, OH), 9.28 (s, 1H, NH), 9.75 (s, 1H, NH); ^13^C-NMR (*d*_6_-DMSO, 101 MHz): δ (ppm) = 27.8, 30.3, 33.1 (CH_2_), 106.7, 114.9 (2C), 121.1 (2C), 121.6, 125.8, 128.3, 158.7 (CH), 132.1, 132.8, 133.7, 141.4, 152.2, 160.4, 162.9, 173.3 (C); C_20_H_18_N_4_O_2_ (346.38): calc. C 69.35, H 5.24 N 16.17, found C 69.54, H 5.26, N 15.86; APCI-MS *m/z* (%) 347.2 [M+H]^+^ (100); HPLC (isocr.): 97.9% at 254 nm, 98.9% at 280 nm, t_ms_ = 2.23 min, t_m_ (DMSO) = 1.02 min (ACN/H_2_O 35:65).

*7-[2-(4-Chloroanilino)pyrimidin-4-yl]-1,3,4,5-tetrahydro-2H-1-benzazepin-2-one (**2f**)*: Synthesis according to General Procedure A with 7-[3-(dimethylamino)acryloyl]-1,3,4,5-tetrahydro-2*H*-benzo[*b*]azepin-2-one (**10**, 103 mg, 0.400 mmol) and *N*-(4-chlorophenyl)guanidinium nitrate (**11f**, 112 mg, 0.480 mmol), reaction time 1 h. Recrystallization from ethanol gave a beige-green powder (70 mg, 48%). m.p.: 268 °C; IR (KBr): ῦ _max_ 3266 (NH), 3188 (NH), 1672 cm^−1^ (C=O); ^1^H-NMR (*d*_6_-DMSO, 400 MHz): δ (ppm) = 2.16–2.23 (m, 4H, CH_2_-CH_2_), 2.75–2.83 (m, 2H, CH_2_), 7.12 (d, *J* = 8.3 Hz, 1H, ArH), 7.35–7.39 (m, 2H, ArH), 7.42 (d, *J* = 5.3 Hz, 1H, pyrimidine-H), 7.86–7.90 (m, 2H, ArH), 8.04 (dd, *J* = 8.3, 2.0 Hz, 1H, ArH), 8.08 (d, *J* = 2.0 Hz, 1H, ArH), 8.54 (d, *J* = 5.2 Hz, 1H, pyrimidine-H), 9.77 (s, 1H, NH), 9.80 (s, 1H, NH); ^13^C-NMR (*d*_6_-DMSO, 126 MHz): δ (ppm) = 27.7, 30.2, 33.1 (CH_2_), 107.9, 120.1, 121.6 (2C), 125.9, 128.3 (2C), 128.4, 158.7 (CH), 124.6, 132.4, 133.7, 139.6, 141.6, 159.8, 163.1, 173.2 (C); C_20_H_17_ClN_4_O (364.83): calc. C 65.84, H 4.70, N 15.36, found C 65.89, H 4.68, N 14.96; APCI-MS *m/z* (%) 365.2 [M+H]^+^ (100); HPLC (isocr.): 97.1% at 254 nm, 97.9% at 280 nm, t_ms_ = 2.47 min, t_m_ (DMSO) = 1.03 min (ACN/H_2_O 60:40).

*7-[2-(4-Bromoanilino)pyrimidin-4-yl]-1,3,4,5-tetrahydro-2H-1-benzazepin-2-one (**2g**)*: Synthesis according to General Procedure A with 7-[3-(dimethylamino)acryloyl]-1,3,4,5-tetrahydro-2*H*-benzo[*b*]azepin-2-one (**10**, 258 mg, 1.00 mmol) and *N*-(4-bromophenyl)guanidinium nitrate (**11g**, 291 mg, 1.05 mmol), reaction time 45 min. Recrystallization from ethanol gave ochre-colored crystals (185 mg, 45%). m.p.: 239 °C; IR (KBr): ῦ _max_ 3258 (NH), 3183 (NH), 1672 cm^−1^ (C=O); ^1^H-NMR (*d*_6_-DMSO, 400 MHz): δ (ppm) = 2.15–2.22 (m, 4H, CH_2_-CH_2_), 2.78–2.86 (m, 2H, CH_2_), 7.11 (d, *J* = 8.3 Hz, 1H, ArH), 7.42 (d, *J* = 5.3 Hz, 1H, pyrimidine-H), 7.47–7.51 (m, 2H, ArH), 8.04 (dd, *J* = 8.2, 2.1 Hz, 1H, ArH), 8.08 (d, *J* = 1.9 Hz, 1H, ArH), 8.54 (d, *J* = 5.3 Hz, 1H, pyrimidine-H), 9.76 (s, 1H, NH), 9.78 (s, 1H, NH); ^13^C-NMR (*d*_6_-DMSO, 101 MHz): δ (ppm) = 27.8, 30.2, 33.1 (CH_2_), 107.9, 120.6 (2C), 121.7, 126.0, 128.4, 131.2 (2C), 158.8 (CH), 112.6, 132.4, 133.8, 140.1, 141.6, 159.8, 163.2, 173.3 (C); C_20_H_17_BrN_4_O (409.28): calc. C 58.69, H 4.19, N 13.69, found C 58.65, H 4.29, N 13.28; APCI-MS *m/z* (%) 409.1 [M+H]^+^ (100); HPLC (isocr.): 97.4% at 254 nm, 97.6% at 280 nm, t_ms_ = 5.66 min, t_m_ (DMSO) = 1.03 min (ACN/H_2_O 65:35).

*7-[2-(4-Iodoanilino)pyrimidin-4-yl]-1,3,4,5-tetrahydro-2H-1-benzazepin-2-one (**2h**)*: Synthesis according to General Procedure A with 7-[3-(dimethylamino)acryloyl]-1,3,4,5-tetrahydro-2*H*-benzo[*b*]azepin-2-one (**10**, 103 mg, 0.400 mmol) and *N*-(4-iodophenyl)guanidinium nitrate (**11h**, 156 mg, 0.480 mmol), reaction time 1.5 h. Recrystallization from ethanol gave violet crystals (122 mg, 67%). m.p.: 275 °C; IR (KBr): ῦ _max_ 3253 (NH), 3177 (NH), 1665 cm^−1^ (C=O); ^1^H-NMR (*d*_6_-DMSO, 400 MHz): δ (ppm) = 2.15–2.22 (m, 4H, CH_2_-CH_2_), 2.78–2.82 (m, 2H, CH_2_), 7.11 (d, *J* = 8.3 Hz, 1H, ArH), 7.41 (d, *J* = 5.3 Hz, 1H, pyrimidine-H), 7.63–7.65 (m, 2H, ArH), 8.04 (dd, *J* = 8.2 Hz, *J* = 2.0 Hz, 1H, ArH), 8.08 (d, *J* = 1.8 Hz, 1H, ArH), 8.54 (d, *J* = 5.3 Hz, 1H, pyrimidine-H), 9.77 and 9.78 (s, 1H, NH and s, 1H, NH; overlapping); ^13^C-NMR(*d*_6_-DMSO, 126 MHz): δ (ppm) = 27.7, 30.2, 33.1 (CH_2_), 107.8, 120.9 (2C), 121.6, 125.9, 128.4, 137.0 (2C), 158.7 (CH), 83.9, 132.3, 133.7, 140.5, 141.6, 159.8, 163.1, 173.2 (C); C_20_H_17_IN_4_O (456.28): calc. C 52.65, H 3.76, N 12.28, found C 52.20, H 3.72, N 11.96; APCI-MS *m/z* (%) 457.1 [M+H]^+^ (100); HPLC (isocr.): 95.1% at 254 nm, 96.8% at 280 nm, t_ms_ = 2.07 min, t_m_ (DMSO) = 1.03 min (ACN/H_2_O 65:35).

*7-[2-(4-Nitroanilino)pyrimidin-4-yl]-1,3,4,5-tetrahydro-2H-1-benzazepin-2-one (**2i**)*: Synthesis according to General Procedure A with 7-[3-(dimethylamino)acryloyl]-1,3,4,5-tetrahydro-2*H*-benzo[*b*]azepin-2-one (**10**, 232 mg, 0.900 mmol) and *N*-(4-nitrophenyl)guanidinium nitrate (**11i**, 243 mg, 1.00 mmol), reaction time 1.5 h. Purification by decocting in 200 mL ethanol gave a brown powder (145 mg, 43%). m.p.: 333 °C; IR (KBr): ῦ _max_ 3271 (NH), 3194 (NH), 1671 cm^−1^ (C=O); ^1^H-NMR (*d*_6_-DMSO, 400 MHz): δ (ppm) = 2.17–2.24 (m, 4H, CH_2_-CH_2_), 2.80–2.84 (m, 2H, CH_2_), 7.14 (d, *J* = 8.3 Hz, 1H, ArH), 7.57 (d, *J* = 5.3 Hz, 1H, pyrimidine-H), 8.07–8.13 (m, 4H, ArH), 8.23–8.27 (m, 2H, ArH), 8.65 (d, *J* = 5.3 Hz, 1H, pyrimidine-H), 9.79 (s, 1H, NH), 10.46 (s, 1H, NH); ^13^C-NMR (*d*_6_-DMSO, 126 MHz): δ (ppm) = 27.7, 30.2, 33.1 (CH_2_), 109.4, 117.6 (2C), 121.7, 125.0 (2C), 126.1, 128.5, 158.9 (CH), 132.0, 133.8, 140.2, 141.8, 147.2, 159.3, 163.4, 173.2 (C); C_20_H_17_N_5_O_3_ (375.38): calc. C 63.99, H 4.56, N 18.66, found C 63.64, H 4.58, N 18.44; APCI-MS *m/z* (%) 376.2 [M+H]^+^ (100); HPLC (isocr.): 99.3% at 254 nm, 99.6% at 280 nm, t_ms_ = 2.97 min, t_m_ (DMSO) = 1.02 min (ACN/H_2_O 50:50).

*7-[2-(2-Hydroxyanilino)pyrimidin-4-yl]-1,3,4,5-tetrahydro-2H-1-benzazepin-2-one (**2j**):* A mixture of 7-[3-(dimethylamino)acryloyl]-1,3,4,5-tetrahydro-2*H*-benzo[*b*]azepin-2-one (**10**, 194 mg, 0.750 mmol), *N*-(2-hydroxyphenyl)guanidinium nitrate (**11j**, 171 mg, 0.800 mmol) and sodium hydroxide (32 mg, 0.80 mmol) was heated in 4 mL propan-2-ol to reflux for 23 h. After cooling to room temperature, a precipitate was formed, which was collected by filtration, washed with water and purified via column chromatography (ethyl acetate: ethanol) to yield a yellow powder (52 mg, 19%). m.p.: 249 °C; IR (KBr): ῦ _max_ 3256 (NH), 3188 (NH), 1666 cm^−1^ (C=O); ^1^H-NMR (*d*_6_-DMSO, 400 MHz): δ (ppm) = 2.12–2.24 (m, 4H, CH_2_-CH_2_), 2.78–2.81 (m, 2H, CH_2_), 6.81–6.91 (m, 3H, ArH), 7.11 (d, *J* = 8.3 Hz, 1H, ArH), 7.40 (d, *J* = 5.3 Hz, 1H, pyrimidine-H), 8.04 (dd, *J* = 8.3, 2.0 Hz, 1H, ArH), 8.08 (d, *J* = 2.0 Hz, 1H, ArH), 8.13 (dd, *J* = 8.1, 2.3 Hz, 1H, ArH), 8.19 (s, 1H, OH), 8.52 (d, *J* = 5.3 Hz, 1H, pyrimidine-H), 9.77 (s, 1H, NH), 10.01 (s, 1H, NH); ^13^C-NMR (*d*_6_-DMSO, 101 MHz): δ (ppm) = 27.8, 30.2, 33.1 (CH_2_),107.5, 115.1, 119.1, 120.3, 121.6, 122.7, 125.9, 128.4, 158.8 (CH),127.9, 132.3, 133.8, 141.6, 146.9, 160.0, 163.3, 173.3 (C); C_20_H_18_N_4_O_2_ (364.38): calc. C 69.35, H 5.24, N 16.17, found C 69.74, H 5.30, N 15.61; HPLC (isocr.): 99.6% at 254 nm, 99.6% at 280 nm, t_ms_ = 4.54 min, t_m_ (DMSO) = 1.03 min (ACN/H_2_O 30:70).

*7-[2-(2-Chloroanilino)pyrimidin-4-yl]-1,3,4,5-tetrahydro-2H-1-benzazepin-2-one (**2k**)*: Synthesis according to General Procedure A with 7-[3-(dimethylamino)acryloyl]-1,3,4,5-tetrahydro-2*H*-benzo[*b*]azepin-2-one (**10**, 232 mg, 0.900 mmol) and *N*-(2-chlorophenyl)guanidinium nitrate (**11k**, 233 mg, 1.00 mmol), reaction time 1.3 h. Recrystallization from ethyl acetate gave a colorless powder (105 mg, 32%). m.p.: 214 °C; IR (KBr): ῦ _max_ 3400 (NH), 3175 (NH), 1672 cm^−1^ (C=O); ^1^H-NMR (*d*_6_-DMSO, 400 MHz): δ (ppm) = 2.14–2.23 (m, 4H, CH_2_-CH_2_), 2.76–2.79 (m, 2H, CH_2_), 7.08 (d, *J* = 8.3 Hz, 1H, ArH), 7.15 („dt“, *J* = 7.8, 1.5 Hz, 1H, ArH), 7.34–7.41 (m, 2H, ArH), 7.52 (dd, *J* = 8.1, 1.5 Hz, 1H, ArH), 7.96–8.00 (m, 2H, ArH), 8.04 (d, *J* = 2.0 Hz, 1H, ArH), 8.48 (d, *J* = 5.3 Hz, 1H, pyrimidine-H), 8.73 (s, 1H, NH), 9.74 (s, 1H, NH); ^13^C-NMR (*d*_6_-DMSO, 101 MHz): δ (ppm) = 27.7, 30.2, 33.1 (CH_2_), 108.0, 121.6, 124.8, 125.1, 125.9, 127.3, 128.4, 129.3, 158.8 (CH), 126.4, 132.3, 133.7, 136.5, 141.6, 160.2, 163.2, 173.2 (C); C_20_H_17_ClN_4_O (364.83): calc. C 65.84, H 4.70, N 15.36, found C 65.69, H 4.74, N 14.95; APCI-MS *m/z* (%) 365.2 [M+H]^+^ (100), 329.3 [M-35] ^+^(26); HPLC (isocr.): 99.6% at 254 nm, 99.7% at 280 nm, t_ms_ = 3.09 min, t_m_ (DMSO) = 1.03 min (ACN/H_2_O 60:40).

*7-[2-(2-Bromoanilino)pyrimidin-4-yl]-1,3,4,5-tetrahydro-2H-1-benzazepin-2-one (**2l**)*: Synthesis according to General Procedure A with 7-[3-(dimethylamino)acryloyl]-1,3,4,5-tetrahydro-2*H*-benzo[*b*]azepin-2-one (**10**, 181 mg, 0.700 mmol) and *N*-(2-bromophenyl)guanidinium nitrate (**11l**, 213 mg, 0.770 mmol), reaction time 55 min. Recrystallization from ethyl acetate gave brown crystals (109 mg, 38%). m.p.: 216 °C; IR (KBr): ῦ _max_ 3413 (NH), 3175 (NH), 1660 cm^−1^ (C=O); ^1^H-NMR (*d*_6_-DMSO, 400 MHz): δ (ppm) = 2.14–2.23 (m, 4H, CH_2_-CH_2_), 2.76–2.78 (m, 2H, CH_2_), 7.07–7.11 (m, 2H, ArH), 7.39–7.44 (m, 2H, ArH), 7.68 (dd, *J* = 8.1, 1.5 Hz, 1H, ArH), 7.93–7.96 and 7.96–7.99 (dd, *J* = 8.1, 1.5 Hz, 1H, ArH and dd, *J* = 8.3, 2.1 Hz, 1H, ArH; overlapping), 8.03 (d, *J* = 2.0 Hz, 1H, ArH), 8.48 (d, *J* = 5.1 Hz, 1H, pyrimidine-H), 8.65 (s, 1H, NH), 9.74 (s, 1H, NH); ^13^C-NMR (*d*_6_-DMSO, 126 MHz): δ (ppm) = 27.7, 30.2, 33.1 (CH_2_), 107.9, 121.5, 125.4, 125.6, 125.8, 127.9, 128.3, 132.3, 158.9 (CH), 117.7, 132.5, 133.6, 137.8, 141.5, 160.2, 163.1, 173.2 (C); C_20_H_17_BrN_4_O (409.28): calc. C 58.69, H 4.19, N 13.69, found C 58.63, H 4.22, N 13.35; APCI-MS *m/z* (%) 410.2 [M+H]^+^ (100); HPLC (isocr.): 99.3% at 254 nm, 99.9% at 280 nm, t_ms_ = 3.49 min, t_m_ (DMSO) = 1.03 min (ACN/H_2_O 60:40).

*7-[2-(3-Hydroxyanilino)pyrimidin-4-yl]-1,3,4,5-tetrahydro-2H-1-benzazepin-2-one (**2m**)*: Synthesis according to General Procedure B with 7-[3-(dimethylamino)acryloyl]-1,3,4,5-tetrahydro-2*H*-benzo[*b*]azepin-2-one (**10**, 129 mg, 0.500 mmol) and *N*-(3-hydroxyphenyl)guanidinium nitrate (**11m**, 107 mg, 0.500 mmol), reaction time 30 min. Recrystallization from ethanol gave beige-colored crystals (66 mg, 38%). m.p.: 243 °C; IR (KBr): ῦ _max_ 3270 (NH), 3191 (NH), 1650 cm^−1^ (C=O); ^1^H-NMR (*d*_6_-DMSO, 400 MHz): δ (ppm) = 2.12–2.26 (m, 4H, CH_2_-CH_2_), 2.76–2.83 (m, 2H, CH_2_), 6.38 (ddd, *J* = 8.1, 2.3, 0.8 Hz, 1H, ArH), 7.06 and 7.12 (t, *J* = 8.1 Hz, 1H, ArH and d, *J* = 8.3 Hz, 1H, ArH; overlapping), 7.20–7.24 (m, 1H, ArH), 7.37 (d, *J* = 5.3 Hz, 1H, pyrimidine-H), 7.41 (t, *J* = 2.3 Hz, 1H, ArH), 8.05 (dd, *J* = 8.1, 2.0 Hz, 1H, ArH), 8.09 (d, *J* = 1.8 Hz, 1H, ArH), 8.52 (d, *J* = 5.3 Hz, 1H, pyrimidine-H), 9.26 (s, 1H, OH), 9.76 (s, 1H, NH); ^13^C-NMR (*d*_6_-DMSO, 101 MHz): δ (ppm) = 27.7, 30.2, 33.1 (CH_2_), 106.0, 107.4, 108.5, 109.8, 121.6, 125.9, 128.4, 129.0, 158.7 (CH), 132.5, 133.7, 141.4, 141.6, 157.4, 160.1, 163.0, 173.2 (C); C_20_H_18_N_4_O_2_ (346.38): calc. C 69.35, H 5.24, N 16.17, found C 68.99, H 5.16, N 15.83; APCI-MS *m/z* (%) 347.2 [M+H]^+^ (100); HPLC (isocr.): 99.8% at 254 nm, 99.9% at 280 nm, t_ms_ = 2.61 min, t_m_ (DMSO) = 1.03 min (ACN/H_2_O 40:60).

*7-[2-(3-Hydroxy-4-methoxyanilino)pyrimidin-4-yl]-1,3,4,5-tetrahydro-2H-1-benzazepin-2-one (**2n**)*: Synthesis according to General Procedure B with 7-[3-(dimethylamino)acryloyl]-1,3,4,5-tetrahydro-2*H*-benzo[*b*]azepin-2-one (**10**, 258 mg, 1.00 mmol) and *N*-(3-hydroxy-4-methoxyphenyl)guanidinium nitrate (**11o**, 244 mg, 1.00 mmol) reaction time 1 h. Recrystallization from ethanol gave brown crystals (136 mg, 36%). m.p.: 269 °C; IR (KBr): ῦ _max_ 3544 (NH), 3280 (NH), 1670 cm^−1^ (C=O); ^1^H-NMR (*d*_6_-DMSO, 400 MHz): δ (ppm) = 2.12–2.26 (m, 4H, CH_2_-CH_2_), 2.75–2.83 (m, 2H, CH_2_), 3.74 (s, 3H, OCH_3_), 6.87 (d, *J* = 8.8 Hz, 1H, ArH), 7.10 (d, *J* = 8.3 Hz, 1H, ArH), 7.18 (dd, *J* = 8.6, 2.5 Hz, 1H, ArH), 7.31 (d, *J* = 5.3 Hz, 1H, pyrimidine-H), 7.37 (d, *J* = 2.5 Hz, 1H, ArH), 8.04 (dd, *J* = 8.3, 2.0 Hz, 1H, ArH), 8.07 (d, *J* = 2.0 Hz, 1H, ArH), 8.47 (d, *J* = 5.3 Hz, 1H, pyrimidine-H), 8.89 (s, 1H, OH), 9.33 (s, 1H, NH), 9.76 (s, 1H, NH); ^13^C-NMR (*d*_6_-DMSO, 101 MHz): δ (ppm) = 56.1 (CH_3_), 27.8, 30.3, 33.1 (CH_2_), 106.9, 107.9, 109.9, 112.8, 121.6, 125.9, 128.4, 158.7 (CH), 132.7, 133.7, 134.3, 142.4, 142.7, 146.4, 160.2, 163.0, 173.3 (C); C_21_H_20_N_4_O_3_ (376.41): calc. C 67.01, H 5.36, N 14.88, found C 66.68, H 5.41, N 14.69; APCI-MS *m/z* (%) 377.2 [M+H]^+^ (100); HPLC (isocr.): 99.9% at 254 nm, 100.0% at 280 nm, t_ms_ = 2.37 min, t_m_ (DMSO) = 1.02 min (ACN/H_2_O 50:50).

*7-{2-[(3-(3-Chloropropoxy)phenyl)amino]pyrimidin-4-yl}-1,3,4,5-tetrahydro-2H-benzo[b]azepin-2-one* (**12**): 7-{2-[(3-Hydroxyphenyl)amino)pyrimidin-4-yl)-1,3,4,5-tetrahydro-2*H*-benzo[*b*]azepin-2-one (**2m**) (199 mg, 0.574 mmol) and cesium carbonate (375 mg, 1.15 mmol) were suspended in ACN (30 mL) and heated for 30 min at reflux. 1-Bromo-3-chloropropane (289 µL, 2.89 mmol) was added dropwise to the hot solution and refluxed for 2 h. After cooling to room temperature the suspension was evaporated and the crude product was purified by column chromatography (ethyl acetate/dichloromethane/triethylamine 3:1:0.1) to obtain a yellow solid (60 mg, 25%). m.p.: 183–185 °C; IR (KBr): ῦ _max_ 3435 (NH), 1671 (C=O) cm^−1^; ^1^H-NMR (*d*_6_-DMSO, 600 MHz): δ (ppm) = 2.13–2.25 (m, 6H, 3 × CH_2_), 2.79 (t, *J* = 7.2 Hz, 2H, CH_2_), 3.82 (t, *J* = 6.5 Hz, 2H, CH_2_), 4.11 (t, *J* = 6.0 Hz, 2H, CH_2_), 6.56 (ddd, *J* = 8.1, 2.5, 1.0 Hz, 1H, ArH), 7.11 (d, *J* = 8.3 Hz, 1H, ArH), 7.20 (t, *J* = 8.1 Hz, 1H, ArH), 7.32 (ddd, *J* = 8.3, 2.0, 0.94 Hz, 1H, ArH), 7.40 (d, *J* = 5.3 Hz, 1H, ArH), 7.72 (t, *J* = 2.3 Hz, 1H, ArH), 8.05 (dd, *J* = 8.2, 2.2 Hz, 1H, ArH), 8.10 (d, *J* = 2.1 Hz, 1H, ArH), 8.54 (d, *J* = 5.3 Hz, 1H, ArH), 9.66 (s, 1H, NH), 9.78 (s, 1H, NH); ^13^C-NMR (*d*_6_-DMSO, 151 MHz): δ (ppm) = 27.7, 30.2, 31.7, 33.1, 42.0, 63.9 (CH_2_), 105.0, 107.0, 107.6, 111.4, 121.6, 125.8, 128.3, 129.2, 158.6 (CH), 132.5, 133.7, 141.5, 141.8, 158.8, 160.0, 163.0, 173.2 (C); C_23_H_23_ClN_4_O_2_ (422.91); APCI-MS: *m/z* (%) = 423 [M+H]^+^ (100).

*7-{2-[(3-(3-Chloropropoxy)-4-methoxyphenyl)amino]pyrimidin-4-yl}-1,3,4,5-tetrahydro-2H-benzo[b]azepin-2-one*: 7-{2-[(3-Hydroxy-4-methoxyphenyl)amino)pyrimidin-4-yl)-1,3,4,5-tetrahydro-2*H*-benzo[*b*]azepin-2-one (**2n**) (100 mg, 0.266 mmol) and cesium carbonate (173 mg, 0.531 mmol) were suspended in ACN (30 mL) and heated for 30 min at reflux. 1-Bromo-3-chloropropane (32 µL, 0.32 mmol) was added dropwise to the hot solution and heated for 4 h to reflux. After cooling to room temperature, the suspension was evaporated and the crude product was purified by column chromatography (ethyl acetate) to obtain a yellow solid (27 mg, 22%). m.p.: 180–182 °C; IR (KBr): ῦ _max_ 3419 and 3287 (NH), 1670 (C=O) cm^−1^; ^1^H-NMR (*d*_6_-DMSO, 600 MHz): δ (ppm) = 2.12–2.25 (m, 6H, 3 × CH_2_), 2.78 (t, *J* = 7.0 Hz, 2H, CH_2_), 3.74 (s, 3H, CH_3_), 3.82 (t, *J* = 6.4 Hz, 2H, CH_2_), 4.10 (t, *J* = 6.0 Hz, 2H, CH_2_), 6.94 (d, *J* = 8.8 Hz, 1H, ArH), 7.10 (d, *J* = 8.2 Hz, 1H, ArH), 7.30 (dd, *J* = 8.8, 2.4 Hz, 1H, ArH), 7.33 (d, *J* = 5.3 Hz, 1H, ArH), 7.66 (s, 1H, ArH), 8.04 (dd, *J* = 8.3, 2.1 Hz, 1H, ArH), 8.06 (d, *J* = 2.1 Hz, 1H, ArH), 8.49 (d, *J* = 5.1 Hz, 1H, ArH), 9.45 (s, 1H, NH), 9.77 (s, 1H, NH); ^13^C-NMR (*d*_6_-DMSO, 151 MHz): δ (ppm) = 56.0 (CH_3_), 27.7, 30.2, 31.8, 33.1, 42.0, 65.0 (CH_2_), 106.0, 107.1, 111.3, 112.7, 121.6, 125.8, 128.2, 158.8 (CH), 132.7, 133.7, 134.3, 141.4, 143.9, 147.5, 160.1, 162.9, 173.2 (C); C_24_H_25_ClN_4_O_3_ (452.94); APCI-MS: *m/z* (%) = 453 [M+H]+ (100).

*7-{2-[(3-(3-Bromopropoxy)phenyl)amino]pyrimidin-4-yl)-1,3,4,5-tetrahydro-2H-benzo[b]azepin-2-one:* 1,3-Dibromopropane (590 µL, 5.79 mmol) was added dropwise to a suspension of 7-{2-[(3-hydroxyphenyl)amino)pyrimidin-4-yl)-1,3,4,5-tetrahydro-2*H*-benzo[*b*]azepin-2-one (**2m**; 201 mg, 0.580 mmol) and anhydrous potassium carbonate (160 mg, 1.16 mmol) in dry acetone (7 mL). Subsequently, the solution was refluxed for 24 h. After cooling, ethyl acetate (15 mL) was added and the solution was washed with water (2 × 20 mL). The organic layer was dried with sodium sulfate and evaporated to dryness. Purification by column chromatography (ethyl acetate/triethylamine 50:1) yielded a yellow solid (135 mg, 50%). m.p.: 182–185 °C; IR (KBr): ῦ _max_ 3283 and 3192 cm^−1^ (NH), 1667 cm^−1^ (C=O); ^1^H-NMR (500 MHz, DMSO-*d*_6_): δ (ppm) = 2.12–2.24 (m, 4H, 2 × CH_2_), 2.27 (quint., *J* = 6.4 Hz, 2H, CH_2_), 2.79 (t, *J* = 7.0 Hz, 2H, CH_2_), 3.69 (t, *J* = 6.5 Hz, 2H, CH_2_), 4.10 (t, *J* = 6.0 Hz, 2H, CH_2_), 6.56 (ddd, *J* = 8.0, 2.5, 0.9 Hz, 1H, ArH), 7.11 (d, *J* = 8.3 Hz, 1H, ArH), 7.20 (t, *J* = 8.2 Hz, 1H, ArH), 7.30–7.37 (m, 1H, ArH), 7.40 (d, *J* = 5.2 Hz, 1H, ArH), 7.72 (t, *J* = 2.3 Hz, 1H, ArH), 8.05 (dd, *J* = 8.3, 2.1 Hz, 1H, ArH), 8.10 (d, *J* = 2.1 Hz, 1H, ArH), 8.54 (d, *J* = 5.3 Hz, 1H, ArH), 9.66 (s, 1H, NH), 9.78 (s, 1H, NH); ^13^C-NMR (126 MHz, *d*_6_-DMSO): δ (ppm) = 27.7, 30.2, 31.3, 31.9, 33.1, 65.0 (CH_2_), 105.0, 107.0, 107.6, 111.4, 121.6, 125.8, 128.3, 129.2, 158.8 (CH), 132.5, 133.7, 141.5, 141.8, 158.6, 160.0, 163.0, 173.2 (C); C_23_H_23_BrN_4_O_2_ (467.37); APCI-MS: *m/z* (%) = 467 [M+H]^+^ (10).

*7-{2-[(3-(3-(5-Methyl-1,1-dioxido-1,2,5-thiadiazolidin-2-yl)propoxy)phenyl)amino]pyrimidin-4-yl}-1,3,4,5-tetrahydro-2H-benzo[b]azepin-2-one* (**14a**): Synthesis according to General Procedure C with 2-methyl-1,2,5-thiadiazolidine-1,1-dioxide (200 mg, 1.47 mmol), sodium hydride (60% dispersion in mineral oil, 72 mg, 1.8 mmol) and 7-{2-[(4-(3-bromopropoxy)phenyl)amino]pyrimidin-4-yl}-1,3,4,5-tetrahydro-2H-benzo[b]azepin-2-one (130 mg, 0.278 mmol) in DMF (9 mL). After a reaction time of 48 h, the product was purified by column chromatography with ethylacetate/dichloromethane (5:1) and obtained as a colorless solid (18 mg, 12%). m.p.: 183–185 °C; IR (KBr): ῦ _max_ 3266 (NH), 1660 (C=O), 1577 cm^−1^; ^1^H-NMR (d_6_-DMSO, 600 MHz): δ (ppm) = 1.97–2.05 (m, 2H, CH_2_), 2.13–2.20 (m, 2H, CH_2_), 2.20–2.25 (m, 2H, CH_2_), 2.60 (s, 3H, CH_3_), 2.79 (t, *J* = 7.0 Hz, 2H, CH_2_), 3.12 (t, *J* = 7.1 Hz, 2H, CH_2_), 3.22–3.28 (m, 2H, CH_2_), 3.29–3.33 (m, 2H, CH_2_), 4.05 (t, *J* = 6.1 Hz, 2H, CH_2_), 6.55 (dd, *J* = 8.1, 2.5 Hz, 1H, ArH), 7.11 (d, *J* = 8.3 Hz, 1H, ArH), 7.20 (t, *J* = 8.1 Hz, 1H, ArH), 7.34 (dd, *J* = 8.2, 2.0 Hz, 1H, ArH), 7.40 (d, *J* = 5.2 Hz, 1H, ArH), 7.67 (t, *J* = 2.3 Hz, 1H, ArH), 8.04 (dd, *J* = 8.2, 2.1 Hz, 1H, ArH), 8.10 (d, *J* = 2.1 Hz, 1H, ArH), 8.54 (d, *J* = 5.2 Hz, 1H, ArH), 9.65 (s, 1H, NH), 9.77 (s, 1H, NH); ^13^C-NMR (d_6_-DMSO, 151 MHz): δ (ppm) = 33.4 (CH_3_), 27.1, 27.7, 30.2, 33.1, 44.4, 45.4, 47.0, 64.5 (CH_2_), 105.1, 107.0, 107.6, 111.3, 121.6, 125.9, 128.3, 129.2, 158.7 (CH), 132.5, 133.7, 141.5, 141.8, 158.8, 160.0, 163.0, 173.2 (C); C_26_H_30_N_6_O_4_S (522.62); HRMS (EI): m/z [M]^+•^ calc. 522.20438, found 522.20448; APCI-MS: m/z (%) = 523 [M+H]^+^ (14); HPLC (isocr.): 99.8% at 254 nm, 99.8% at 280 nm, t_ms_ = 8.29 min, t_m_ (DMSO) = 1.21 min (ACN/H_2_O 50:50); HPLC (gradient): 98.8% at 254 nm, t_ms_ = 10.3 min, t_m_ = 1.17 min; λ_max_ 212, 281 nm.

*N-{3-[3-((4-(2-Oxo-2,3,4,5-tetrahydro-1H-benzo[b]azepin-7-yl)pyrimidin-2-yl)amino)phenoxy]-propyl}morpholine-4-sulfonamide* (**14b**): According to General Procedure D, potassium carbonate (196 mg, 1.42 mmol), morpholine-4-sulfonamide (236 mg, 1.42 mmol) and sodium iodide (26 mg, 0.17 mmol) were added to a solution of 7-{2-[(3-(3-chloropropoxy)phenyl)amino]pyrimidin-4-yl}-1,3,4,5-tetrahydro-2*H*-benzo[*b*]azepin-2-one (**12**) (120 mg, 0.284 mmol) in DMF (9 mL). The resulting yellow suspension was heated at 80 °C for 51 h. The crude product was purified by preparative HPLC (ACN/H_2_O 40:60) to yield a colorless solid (22 mg, 14%); m.p.: 221 °C (discoloration starting at 102 °C); IR (KBr): ῦ _max_ 3429 (NH), 1667 (C=O), 1578 cm^−1^; ^1^H-NMR (*d*_6_-DMSO, 600 MHz): δ (ppm) = 1.92 (quint., *J* = 6.8 Hz, 2H, CH_2_), 2.14–2.25 (m, 4H, 2 × CH_2_), 2.79 (t, *J* = 7.0 Hz, 2H, CH_2_), 2.99 (t, *J* = 4.8 Hz, 4H, 2 × CH_2_), 3.10 (q, *J* = 7.0 Hz, 2H, CH_2_), 3.59 (t, *J* = 4.8 Hz, 4H, 2 × CH_2_), 4.03 (t, *J* = 6.1 Hz, 2H, CH_2_), 6.54 (dd, *J* = 8.1, 2.5 Hz, 1H, ArH), 7.11 (d, *J* = 8.3 Hz, 1H, ArH), 7.20 (t, *J* = 8.1 Hz, 1H, ArH), 7.33–7.36 (m, 1H, ArH), 7.38–7.43 (m, 2H, ArH und NH), 7.68 (t, *J* = 2.3 Hz, 1H, ArH), 8.04 (dd, *J* = 8.3, 2.1 Hz, 1H, ArH), 8.11 (d, *J* = 2.3 Hz, 1H, ArH), 8.54 (d, *J* = 5.3 Hz, 1H, ArH), 9.65 (s, 1H, NH), 9.77 (s, 1H, NH); ^13^C-NMR (*d*_6_-DMSO, 151 MHz): δ (ppm) = δ (ppm) = 27.7, 29.1, 30.2, 33.1, 39.4 (overlapping with DMSO peak), 45.7 (2 C), 64.3, 65.4 (2 C) (CH_2_), 105.1, 107.0, 107.6, 111.2, 121.6, 125.9, 128.3, 129.1, 158.7 (CH), 132.5, 133.7, 141.5, 141.8, 158.8, 160.0, 163.0, 173.2 (C); C_27_H_32_N_6_O_5_S (552.65); HRMS (ESI): *m/z* (C_27_H_33_N_6_O_5_S, monocation) calc. 553.22277, found 553.22303; MS (ESI, positive): *m/z* (%) = 553 [M]^+^ (100), 466 [M^+^–86] (19), 381 [M^+^ –171] (25); HPLC (isocr.): 99.4% at 254 nm, 99.2% at 280 nm, t_ms_ = 6.70 min, t_m_ (DMSO) = 1.20 min (ACN/H_2_O 40:60); HPLC (gradient): 97.5% at 254 nm, *t*_ms_ = 17.5 min, *t*_m_ = 1.40 min; λ_max_ 216, 278 nm.

*N-{3-[3-((4-(2-Oxo-2,3,4,5-tetrahydro-1H-benzo[b]azepin-7-yl)pyrimidin-2-yl)amino)phenoxy]propyl}-pyrrolidine-1-sulfonamide* (**14c**): According to General Procedure D, potassium carbonate (131 mg, 0.948 mmol), pyrrolidine-4-sulfonamide (179 mg, 1.19 mmol) and sodium iodide (23 mg, 0.15 mmol) were added to a solution of 7-{2-[(3-(3-chloropropoxy)phenyl)amino]pyrimidin-4-yl}-1,3,4,5-tetrahydro-2*H*-benzo[b]azepin-2-one (**12**) (100 mg, 0.237 mmol) in DMF (9 mL). The resulting yellow suspension was heated at 80 °C for 22 h. The crude product was purified by preparative HPLC (ACN/H_2_O 40:60) to yield a colorless solid (20 mg, 16%); m.p.: 225 °C (discoloration starting at 100 °C); IR (KBr): ῦ _max_ 3434 (NH), 1667 (C=O), 1578 cm^−1^; ^1^H-NMR (*d*_6_-DMSO, 600 MHz): δ (ppm) = 1.73–1.82 (m, 4H, 2 × CH_2_), 1.91 (quint., *J* = 6.9 Hz, 2H, CH_2_), 2.13–2.25 (m, 4H, 2 × CH_2_), 2.79 (t, *J* = 7.2 Hz, 2H, CH_2_), 3.09 (q, *J* = 7.0 Hz, 2H, CH_2_), 3.11–3.15 (m, 4H, 2 × CH_2_), 4.03 (t, *J* = 6.1 Hz, 2H, CH_2_), 6.54 (ddd, *J* = 8.1, 2.5, 1.0 Hz, 1H, ArH), 7.11 (d, *J* = 8.5 Hz, 1H, ArH), 7.15 (t, *J* = 5.9 Hz, 1H, NH), 7.19 (t, *J* = 8.2 Hz, 1H, ArH), 7.32 (ddd, *J* = 8.3, 2.1, 0.9 Hz, 1H, ArH), 7.40 (d, *J* = 5.3 Hz, 1H, ArH), 7.68 (t, *J* = 2.4 Hz, 1H, ArH), 8.04 (dd, *J* = 8.3, 2.1 Hz, 1H, ArH), 8.11 (d, *J* = 2.3 Hz, 1H, ArH), 8.54 (d, *J* = 5.1 Hz, 1H, ArH), 9.65 (s, 1H, NH), 9.76 (s, 1H, NH); ^13^C-NMR (*d*_6_-DMSO, 151 MHz): δ (ppm) = 24.9 (2C), 27.7, 29.0, 30.2, 33.1, 39.4 (overlapping with DMSO peak), 47.5 (2C), 64.4 (CH_2_), 105.0, 107.0, 107.6, 111.2, 121.6, 125.9, 128.3, 129.1, 158.8 (CH), 132.5, 133.7, 141.5, 141.8, 158.8, 160.0, 163.0, 173.2 (C); C_27_H_32_N_6_O_4_S (536.65); HRMS (ESI): *m/z* (C_27_H_33_N_6_O_4_S, monocation) calc. 537.22785, found 537.22812; MS (ESI, positive): *m/z* (%) = 537 [M+H]^+^ (100), 381 [M^+^ −155] (15), 466 [M^+^ −70] (11); HPLC (isocr.): 99.6% at 254 nm, 99.9% at 280 nm, t_ms_ = 10.3 min, t_m_ (DMSO) = 1.20 min (ACN/H_2_O 40:60); HPLC (gradient): 99.6% at 254 nm, *t*_ms_ = 10.6 min, *t*_m_ = 1.23 min; λ_max_ 213, 280 nm.

*N-{3-[3-((4-(2-Oxo-2,3,4,5-tetrahydro-1H-benzo[b]azepin-7-yl)pyrimidin-2-yl)amino)phenoxy]propyl}-azetidine-1-sulfonamide* (**14d**): According to General Procedure D, potassium carbonate (157 mg, 0.1.14 mmol), azetidine-1-sulfonamide (193 mg, 1.42 mmol) and sodium iodide (26 mg, 0.17 mmol) were added to a solution of *7-{2-[(3-(3-Chloropropoxy)phenyl)amino]pyrimidin-4-yl}-1,3,4,5-tetrahydro-2H-benzo[b]azepin-2-one* (**12**) (121 mg, 0.285 mmol) in DMF (9 mL). The resulting yellow suspension was heated at 80 °C for 22 h. The crude product was purified by preparative HPLC (ACN/H_2_O 40:60) to yield a colorless solid (50 mg, 34%); m.p.: 248 °C (discoloration starting at 115 °C); IR (KBr): ῦ _max_ 3280 (NH), 1668 (C=O), 1579 cm^−1^; ^1^H-NMR (*d*_6_-DMSO, 500 MHz): δ (ppm) = 1.92 (quint., *J* = 6.6 Hz, 2H, CH_2_), 2.01–2.11 (m, 2H, CH_2_), 2.12–2.26 (m, 4H, 2 × CH_2_), 2.79 (t, *J* = 7.0 Hz, 2H, CH_2_), 3.12 (q, *J* = 7.0 Hz, 2H, CH_2_), 3.68 (t, *J* = 7.7 Hz, 4H, 2 × CH_2_), 4.03 (t, *J* = 6.1 Hz, 2H, CH_2_), 6.55 (ddd, *J* = 8.2, 2.4, 0.9 Hz, 1H, ArH), 7.11 (d, *J* = 8.2 Hz, 1H, ArH), 7.20 (t, *J* = 8.2 Hz, 1H, ArH), 7.24 (t, *J* = 6.0 Hz, 1H, ArH), 7.30–7.36 (m, 1H, NH), 7.39 (d, *J* = 5.2 Hz, 1H, ArH), 7.68 (t, *J* = 2.1 Hz, 1H, ArH), 8.04 (dd, *J* = 8.2, 2.1 Hz, 1H, ArH), 8.11 (d, *J* = 2.1 Hz, 1H, ArH), 8.54 (d, *J* = 5.2 Hz, 1H, ArH), 9.64 (s, 1H, NH), 9.76 (s, 1H, NH); ^13^C-NMR (*d*_6_-DMSO, 126 MHz): δ (ppm) = 14.4, 27.7, 29.2, 30.2, 33.1, 39.4 (overlapping with DMSO peak), 49.7 (2C), 64.4 (CH_2_), 105.0, 107.0, 107.6, 111.2, 121.6, 125.9, 128.3, 129.1, 158.8 (CH), 132.5, 133.7, 141.5, 141.8, 158.8, 160.0, 163.0, 173.2 (C) (the signal at 158.8 ppm corresponds to one quaternary and one tertiary carbon atom); C_26_H_30_N_6_O_4_S (522.62); HRMS (EI): *m/z* [M]^+•^ calc. 522.20438,found 522.20251; MS (ESI, positive): *m/z* (%) = 523 [M]^+^ (100), 466 [M^+^–56] (18), 381 [M^+^–141] (5); HPLC (isocr.): 96.9% at 254 nm, 99.8% at 280 nm, t_ms_ = 7.68 min, t_m_ (DMSO) = 1.20 min (ACN/H_2_O 40:60); HPLC (gradient): 99.1% at 254 nm, *t*_ms_ = 10.2 min, *t*_m_ = 1.27 min; λ_max_ 212, 281 nm.

*7-(2-((4-Methoxy-3-(3-(5-methyl-1,1-dioxido-1,2,5-thiadiazolidin-2-yl)propoxy)phenyl)amino)pyrimidin-4-yl)-1,3,4,5-tetrahydro-2H-benzo[b]azepin-2-one* (**14e**): According to General Procedure C, with 2-methyl-1,2,5-thiadiazolidine-1,1-dioxide (142 mg, 1.04 mmol), sodium hydride (60% dispersion in mineral oil, 50 mg, 1.3 mmol) and 7-{2-[(3-(3-chloropropoxy)-4-methoxyphenyl)amino]pyrimidin-4-yl}-1,3,4,5-tetrahydro-2H-benzo[b]azepin-2-one (69 mg, 0.15 mmol) in DMF (9 mL). After a reaction time of 48 h, the product was purified by column chromatography with ethylacetate/ethanol (1:0 → 0.75:0.25 → 0.5:0.5→ 0:1). After recrystallization with ethanol the product was obtained as a yellow solid (20 mg, 24%); m.p.: 198–201 °C; IR (KBr): ῦ _max_ 3264 (NH), 1670 (C=O), 1578 cm^−1^; ^1^H-NMR (d_6_-DMSO, 600 MHz): δ (ppm) = 2.02 (quint., *J* = 6.4 Hz, 2H, CH_2_), 2.10–2.26 (m, 4H, 2 × CH_2_), 2.59 (s, 3H, CH_3_), 2.78 (t, *J* = 7.0 Hz, 2H, CH_2_), 3.12 (t, *J* = 7.1 Hz, 2H, CH_2_), 3.22–3.27 (m, 2H, CH_2_), 3.28–3.33 (m, 2H, CH_2_), 3.74 (s, 3H, OCH_3_), 4.04 (t, *J* = 6.1 Hz, 2H, CH_2_), 6.93 (d, *J* = 8.9 Hz, 1H, ArH), 7.10 (d, *J* = 8.3 Hz, 1H, ArH), 7.31 (d, *J* = 2.4 Hz, 1H, ArH), 7.32–7.34 (m, 1H, ArH), 7.61 (d, *J* = 2.4 Hz, 1H, ArH), 8.03 (dd, *J* = 8.2, 2.1 Hz, 1H), 8.07 (d, *J* = 2.2 Hz, 1H, ArH), 8.48 (d, *J* = 5.2 Hz, 1H, ArH), 9.44 (s, 1H, NH), 9.76 (s, 1H, NH); ^13^C-NMR (d_6_-DMSO, 151 MHz): δ (ppm) = 33.4, 56.0 (CH_3_), 27.2, 27.7, 30.2, 33.1, 44.5, 45.3, 47.0, 65.6 (CH_2_), 105.9, 107.0, 111.1, 112.7, 121.6, 125.8, 128.2, 158.8 (CH), 132.7, 133.7, 134.3, 141.4, 143.9, 147.7, 160.1, 162.9, 173.2 (C); C_27_H_32_N_6_O_5_S (552.65); APCI-MS: m/z (%) = 553 [M+H]^+^ (18); HRMS (EI): m/z [M]^+•^ calc. 552.21494, found 552.21475; HPLC (isocr.): 98.2% at 254 nm, 97.3% at 280 nm, t_ms_ = 5.35 min, t_m_ (DMSO) = 1.19 min (ACN/H_2_O 40:60); HPLC (gradient): 95.6% at 254 nm, t_ms_ = 9.81 min, t_m_ = 1.16 min; λ_max_ 298 nm.

*N-{3-[2-Methoxy-5-((4-(2-oxo-2,3,4,5-tetrahydro-1H-benzo[b]azepin-7-yl)pyrimidin-2-yl)amino)phenoxy]-propyl}morpholine-4-sulfonamide* (**14f**): According to General Procedure D, potassium carbonate (229 mg, 1.66 mmol), morpholine-4-sulfonamide (275 mg, 1.66 mmol) and sodium iodide (30 mg, 0.20 mmol) were added to a solution of 7-(2-((3-(3-chloropropoxy)-4-methoxyphenyl)amino)pyrimidin-4-yl)-1,3,4,5-tetrahydro-2H-benzo[b]azepin-2-one (150 mg, 0.331 mmol) in DMF (9 mL). The resulting yellow suspension was heated at 80 °C for 23 h. The crude product was purified by preparative HPLC (ACN/H_2_O 40:60) to yield a colorless solid (12 mg, 6%). m.p.: 176 °C (discoloration starting at 100 °C); IR (KBr): ῦ _max_ 3433 (NH), 1672 (C=O), 1578 cm^−1^; ^1^H-NMR (d_6_-DMSO, 600 MHz): δ (ppm) = 1.93 (quint., *J* = 6.4 Hz, 2H, CH_2_), 2.12–2.25 (m, 4H, 2 × CH_2_), 2.78 (t, *J* = 7.1 Hz, 2H, CH_2_), 2.97–3.02 (m, 4H, 2 × CH_2_), 3.11 (q, *J* = 7.0 Hz, 2H, CH_2_), 3.57–3.61 (m, 4H, 2 × CH_2_), 3.74 (s, 3H, OCH_3_), 4.04 (t, *J* = 6.0 Hz, 2H, CH_2_), 6.92 (d, *J* = 8.8 Hz, 1H, ArH), 7.10 (d, *J* = 8.3 Hz, 1H, ArH), 7.29 (dd, *J* = 8.8, 2.5 Hz, 1H, ArH), 7.33 (d, *J* = 5.3 Hz, 1H, ArH), 7.35 (t, *J* = 5.7 Hz, 1H, NH), 7.64 (s, 1H, ArH), 8.03 (dd, *J* = 8.3, 2.3 Hz, 1H, ArH), 8.08 (d, *J* = 2.3 Hz, 1H, ArH), 8.48 (d, *J* = 5.1 Hz, 1H, ArH), 9.45 (s, 1H, NH), 9.75 (s, 1H, NH); ^13^C-NMR (d_6_-DMSO, 151 MHz): δ (ppm) = 55.9 (CH_3_), 27.7, 29.1, 30.2, 33.1, 39.4 (overlapping with DMSO peak), 45.7 (2C), 65.4 (2C), 65.5 (CH_2_), 105.6, 107.0, 111.0, 112.6, 121.6, 125.8, 128.2, 158.8 (CH), 132.7, 133.7, 134.3, 141.4, 143.8, 147.6, 160.1, 162.9, 173.2 (C); C_28_H_34_N_6_O_6_S (582.68); HRMS (ESI): m/z (C_28_H_35_N_6_O_6_S, monocation) calc. 583.23333, found 583.23362; MS (ESI, positive): m/z (%) = 583 [M]^+^ (100), 381 [M^+^–201] (37), 102 [M^+^–480] (74); HPLC (isocr.): 94.8% at 254 nm, 95.1% at 280 nm, t_ms_ = 5.43 min, t_m_ (DMSO) = 1.20 min (ACN/H_2_O 40:60); HPLC (gradient): 95.9% at 254 nm, t_ms_ = 9.77 min, t_m_ = 1.27 min; λ_max_ 289 nm.

*N-{3-[2-Methoxy-5-((4-(2-oxo-2,3,4,5-tetrahydro-1H-benzo[b]azepin-7-yl)pyrimidin-2-yl)amino)phenoxy]-propyl}azetidine-1-sulfonamide* (**14g**): According to General Procedure D, potassium carbonate (95 mg, 0.69 mmol), azetidine-1-sulfonamide (117 mg, 0.859 mmol) and sodium iodide (16 mg, 0.11 mmol) were added to a solution of 7-(2-((3-(3-chloropropoxy)-4-methoxyphenyl)amino)pyrimidin-4-yl)-1,3,4,5-tetrahydro-2H-benzo[b]azepin-2-one (78 mg, 0.17 mmol) in DMF (9 mL). The resulting yellow suspension was heated at 80 °C for 48 h. The crude product was purified by preparative HPLC (ACN/H_2_O 40:60) to yield a colorless solid (12 mg, 6%); m.p.: 167–170 °C; IR (KBr): ῦ _max_ 3279 (NH), 1669 (C=O), 1578 cm^−1^; ^1^H-NMR (d_6_-DMSO, 600 MHz): δ (ppm) = 1.93 (quint., *J* = 6.6 Hz, 2H, CH_2_), 2.02–2.10 (m, 2H, CH_2_), 2.13–2.26 (m, 4H, 2 × CH_2_), 2.78 (t, *J* = 7.0 Hz, 2H, CH_2_), 3.13 (q, *J* = 7.0 Hz, 2H, CH_2_), 3.68 (t, *J* = 7.4 Hz, 4H, 2 × CH_2_), 3.74 (s, 3H, OCH_3_), 4.04 (t, *J* = 6.1 Hz, 2H, CH_2_), 6.92 (d, *J* = 8.9 Hz, 1H, ArH), 7.10 (d, *J* = 8.3 Hz, 1H, ArH), 7.19 (t, *J* = 5.8 Hz, 1H, NH), 7.29 (dd, *J* = 8.8, 2.5 Hz, 1H, ArH), 7.33 (d, *J* = 5.3 Hz, 1H, ArH), 7.65 (s, 1H, ArH), 8.03 (dd, *J* = 8.3, 2.3 Hz, 1H, ArH), 8.08 (d, *J* = 2.3 Hz, 1H, ArH), 8.48 (d, *J* = 5.3 Hz, 1H, ArH), 9.45 (s, 1H, NH), 9.75 (s, 1H, NH); ^13^C-NMR (d_6_-DMSO, 151 MHz): δ (ppm) = 55.9 (CH_3_), 14.4, 27.7, 29.2, 30.2, 33.1, 39.4 (overlapping with DMSO peak), 49.7 (2C), 65.5 (CH_2_), 105.6, 107.0, 111.0, 112.6, 121.6, 125.8, 128.2, 158.8 (CH), 132.7, 133.7, 134.3, 141.4, 143.8, 147.7, 160.1, 162.9, 173.2 (C); C_27_H_32_N_6_O_5_S (552.65); HRMS (EI): m/z [M]^+•^ calc. 552.21494, found 552.21431; APCI-MS: m/z (%) = 553 [M+H]^+^ (7); HPLC (isocr.): 97.9% at 254 nm, 98.9% at 280 nm, t_ms_ = 6.20 min, t_m_ (DMSO) = 1.29 min (ACN/H_2_O 40:60); HPLC (gradient): 96.6% at 254 nm, t_ms_ = 9.98 min, t_m_ = 1.17 min; λ_max_ 289 nm.

### 4.3. Molecular Docking

Docking studies were performed using GOLD [25]. The protein and the ligands were prepared using MOE (Molecular Operating Environment, 2018.01, Chemical Computing group, Montreal, QC, Canada). Energy minimization was performed using the QuickPrep function in MOE and structures were saved as mol2 files. Docking runs were performed using the wizard of GOLD in the HERMES interface (version 1.6.2). Missing hydrogen atoms were added, and the ligand and all water molecules were removed from the protein structure. The binding site was defined as a zone of 10 Å around the cocrystallized inhibitor. For scoring, the chemscore kinase function was applied. Docking accuracy was set to 200%, 10 docking runs for each ligand were performed, the function “generate diverse solutions” was activated, and the option “allow early termination” was turned off. The results of the docking experiments were analyzed and visualized using UCSF Chimera (version 1.11.2) [54].

### 4.4. Crystallization of Aurora A Complexes, Data Collection and Structure Determination

Crystallizations of the Aurora A complexes were carried out as described previously [55]. In brief, the recombinant kinase domain of Aurora A was mixed with the compounds, and crystallization was performed at 4 °C using the conditions containing 18% PEG 3350 and 5% tacsimate pH 7 (for **2a**) or 24% PEG 3350 and 0.2 M sodium malonate pH 7. Diffraction data collected at SLS beamline X06SA were processed using XDS [56] and scaled with AIMLESS [57]. Molecular replacements were performed using Phaser [58] and the coordinates of Aurora A (PDB 5ONE) [55]. Model rebuilding was performed in COOT [59], alternated with refinement in REFMAC [60]. The final structures were validated for geometric correctness with MolProbity [61], and were deposited under PDB accession codes 7AYI and 7AYH.

### 4.5. Protein Kinase Inhibition Assays

The screening for protein kinase inhibition was performed with the radiometric ^33^Panqinase activity assay at Reaction Biology Europe GmbH (Freiburg) using a robotic system (Beckmann Coulter/SagianRoboter) and 96-well plates (FlashPlates, Perkin/Elmer/NEN, Boston, MA, USA). In brief, the transfer of ^33^P-phosphate into the substrate was measured with a microplate scintillation counter (Microbeta Trilux, Wallace). Protein kinases were expressed in Sf9 cells as recombinant glutathione *S*-transferase (GST) or polyhistidine (His)-tagged proteins in baculovirus. Isolation and purification of expressed kinases were performed with affinity chromatography by using GSH agarose (Sigma) or Ni-NTA agarose (Qiagen). The standard solution for each enzyme contained: 60 mM HEPES-NaOH (pH 7.5), 3 mM MgCl_2_, 3 µM sodium orthovanadate, 1.2 mM DTT, 50 µg/mL PEG_20000_ and 1 µM [γ-^33^P]-ATP (approximately 5 × 10^5^ cpm per well). In chronological order the reaction mixture was prepared: 20 µL standard solution, 5 µL ATP solution in water, 5 µL of the compound in 10% DMSO and 20 µL of enzyme/substrate mixture (50:50). Each kinase contained different substrates in different concentrations: 125 ng/50 µL Poly(Glu, Tyr)_4:1_ for 20 ng/50 µL IGF1-R, 25 ng/50 µL IGF1-R, 20 ng/50 µL EGF-R, 25 ng/50 µL EGF-R, 40 ng/50 µL EGF-R, 10 ng/50 µL EPHB4, 20 ng/50 µL EPHB4, 200 ng/50 µL ERBB2, 10 ng/50 µL SRC, 100 ng/50 µL FAK, 200 ng/50 µL FAK, 50 ng/50 µL VEGF-R2, 100 ng/50 µL VEGF-R3, 200 ng/50 µLTIE2; 250 ng/50 µL Poly(Glu, Tyr)_4:1_ for 200 ng/50 µL TIE2; 500 ng/50 µL tetra (LRRWSLG) for 50 ng/50 µL Aurora-A, 25 ng/50 µL PAK4, 25 ng/50 µL PDK1; 250 ng/50 µL tetra (LRRWSLG) for 50 ng/50 µL Aurora-B, 200 ng/50 µL Aurora-B; 125 ng/50 µL Histon H1 for 100 ng/50 µL CDK2/CycA; 250 ng/50 µL Rb-CTF for 50 ng/50 µL CDK/CycD1; 500 ng/50 µL Rb-CTF for 50 ng/50 µL CDK4/CycD1; 125 ng/50 µL Poly(ala, Glu, Lys, Tyr)_6:2:5:1_ for 25 ng/50 µL INS-R, 50 ng/50 µL PDGFR-beta, 100 ng/50 µL PDGFR-beta, 100 ng/50 µL FLT3, 150 ng/50 µL FLT3, 20 ng/50 µL MET, 50 ng/50 µL MET, 100 ng/50 µL MET; 250 ng/50 µL Casein for 200 ng/50 µL CK2-alpha1, 200 ng/50 µL PLK1; 1000 ng/50 µL Casein for 200 ng/50 µL PLK1, 200 ng/50 µL CK2-alpha1; 1000 ng/50 µLGSK3 (14–27) for 100 ng/50 µL AKT1; 125 ng/50 µL p53-CTM for 150 ng/50 µL CK2-alpha1, 200 ng/50 µLCK2-alpha1; 250 ng/50 µL MEK-KM for 20 ng/50 µL B-RAF-VE; autophosphorylation at ARK5, COT and SAK. After incubation (80 min, 30 °C) the reaction was stopped by adding 50 µL 2% (*v/v*) phosphoric acid to the reaction mixture. The microplate was washed twice with 200 µL 0.9% (*v/v*) saturated sodium chloride or water. The measured radioactivity, conditioned by the incorporation of [γ-^33^P]-phosphate, is proportional to kinase activity. Furthermore, low control and high control were measured to determine the residual activity (%). As low controls, mixtures with substrate, but without enzyme were used to determine the unspecific binding of [γ-^33^P]-phosphate to the well plate. Mixtures with enzyme, but without inhibitor were used as high controls to determine maximum enzyme activity. The IC_50_-values of the distinct compounds were calculated with Quattro Workflow V1.1.0.8 (Quattro Research GmbH, München, Germany). Dose response curves were generated by measurement of the residual activity of ten different inhibitor concentrations (0.1 mM, 30.0 µM, 10 µM, 3.0 µM, 1.0 µM, 0.3 µM, 0.1 µM, 30.0 nM, 10.0 nM and 3.0 nM). The residual activity was calculated using Equation (1):

(1) $$\mathrm{residual}\;\mathrm{activity}\;\left(\%\right)=100\times \frac{\left(\mathrm{cpm}\;\mathrm{compound}\;-\;\mathrm{low}\;\mathrm{control}\right)}{\left(\mathrm{high}\;\mathrm{control}\;-\;\mathrm{low}\;\mathrm{control}\right)}$$

### 4.6. Kinetic Solubility

The kinetic solubility of test compounds was determined by nephelometry. Dilution series of test compounds in DMSO with eight to ten different concentrations were prepared. The dilutions were pipetted (2 µL) into the cavities of a 96 well plate (CorningTM UV-transparent flat-bottom microtiter plate; Corning Incorporated, Kennebunk, ME, USA). A phosphate buffer solution pH 7.4 (198 µL) was added, so that the DMSO concentration in the measured solutions was 1%. Three wells were filled with DMSO and phosphate buffer pH 7.4 (2:198) and measured as blank values. The 96 well plate prepared in this way was measured with the NEPHELOstar^®^ Plus nephelometer (BMG Labtech GmbH, Ortenberg, Germany, software: Omega 5.11) at a temperature of 25 °C. Previously, the plate was shaken at 500 rpm for 10 s and the laser intensity was set to 80%. The beam focus was set to 2.50 mm. If the test substance is completely soluble, only a scattering close to the blank value is detected. As soon as a critical concentration is reached in which the kinetic solubility of the test compound is exceeded, solid particles precipitate. Undissolved particles in the sample scatter the laser beam. The intensity of the scattered light is determined, which is proportional to the number of particles in the solution. The blank values were determined and subtracted from the measured values of the test compounds. By measuring different concentrations of the test substances, different intensities of the scattered light, indicated as RNU (relative nephelometric units), were determined. Kick off curves were obtained by plotting concentrations against the RNU values. The point of intersection of the horizontal and of the ascending part of the kick-off curve indicated the kinetic solubility concentration.

## 5. Conclusions

The new chemotype **2** was created from the dual PLK1/VEGF-R2 inhibitor **1** by the “cut and glue” method, i.e., formal cutting the structure and joining at different molecular locations. The syntheses of the new structures were performed by standard methods, a key step being the reaction of an enaminone with aromatic guanidium nitrates. The prototype **2a** turned out to be an inhibitor of the protein kinases Aurora A, VEGF-R2 and VEGF-R3. Based on the results of X-ray structure analyses and docking experiments, **2a** was chemically modified by attachment of substituents to the aniline ring. The most potent of the resulting compounds exhibited inhibition of the Aurora A kinase in the single-digit micromolar concentration range. The additional attachment of sulfamide structures via propoxy linkers led to analogues with retained protein kinase inhibitory activity, but reduced the solubility. Two representatives of the new substance class showed very interesting antiproliferative activity in the NCI in vitro cell line screening, justifying a further structural optimization of the new chemotype. A further optimization based on the X-ray structures presented here should focus on modifications of the molecular regions that potentially form additional interactions in the center of the binding pocket.

## Figures and Tables

**Table 1:** Data collection and refinement statistics of the Aurora A complex with ligands **2a** and **2c**.

PDB Accession Code	2a 7AYI	2c 7AYH
	Data collection	Data collection
beamline	Swiss light source X06SA	Swiss light source X06SA
Resolution ^a^ (Å)	44.43–2.86 (3.01–2.86)	44.28–2.80 (2.95–2.80)
space group	*P* 6_1_22	*P* 6_1_22
cell dimensions		
a (Å)	82.6	83.4
b (Å)	82.6	83.4
c (Å)	170.1	168.2
α (deg)	90	90
β (deg)	90	90
γ (deg)	120	120
no. unique reflections ^a^	8526 (1186)	9066 (1279)
Completeness ^a^ (%)	100.0 (100.0)	99.9 (100.0)
I/σI ^a^	25.7 (2.1)	19.3 (2.1)
R_merge_^a^	0.038 (0.975)	0.046 (0.968)
CC(1/2)	1.000 (0.797)	0.999 (0.818)
Redundancy ^a^	8.4 (8.6)	8.5 (9.0)
no. atoms in refinement (P/L) ^b^	2014/25	2004/27
R_fact_ (%)	25.2	27.3
R_free_ (%)	29.7	31.3
Bf (P/L/O) ^b^ (Å^2^)	121/115	133/112
rmsd bond ^c^ (Å)	0.011	0.011
rmsd deviation angle ^c^ (deg)	0.96	1.14
Ramachandran favored	94.35	95.12
Ramachandran outlier	0	0

^a^ Values in brackets show the statistics for the highest resolution shells. ^b^ P/L indicate protein and ligand molecule respectively. ^c^ rms indicates root-mean-square deviation.

**Table 2:**
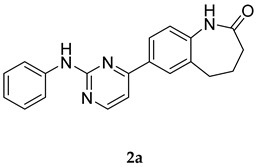
Inhibition profile of **2a** against a panel of cancer-related protein kinases ^a^.

Protein Kinase	IC_50_ (µM)	Protein Kinase	IC_50_ (µM)
**AKT1**	>100	**IGF1-R**	>100
**ARK5**	>100	**INS-R**	>100
**Aurora-A**	2.4	**MET**	>100
**Aurora-B**	26	**PAK4**	>100
**CDK2/CycA**	60	**PDGFR-beta**	44
**CDK4/CycD1**	19	**PDK1**	>100
**CK2-alpha1**	>100	**PLK1**	>100
**EGF-R**	>100	**SAK**	>100
**EPHB4**	>100	**SRC**	74
**ERBB2**	>100	**TIE2**	19
**FAK**	>100	**VEGF-R2**	2.8
**FLT3**	16	**VEGF-R3**	5.9

^a^ As a measure of assay quality, the Z’-factor [26,27] was used. The Z’ factor did not drop below 0.4 and was above 0.6 in most cases, indicating a very good to excellent assay quality.

**Table 3:**
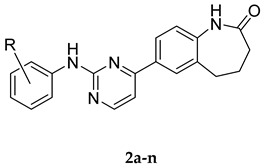
Kinase inhibitory activity of 7-(2-anilinopyrimidin-4-yl)-1-benzazepin-2-ones **2a**–**2n** against Aurora A, Aurora B, VEGF-R2 and VEGF-R3, represented by either IC_50_ value (µM) or residual activity at 10 µM (%) ^a^.

Compound	R	Aurora A	Aurora B	VEGF-R2	VEGF-R3
**2a**	H	2.4 µM	26 µM	2.8 µM	5.9 µM
**2b**	4-CH_3_	2.4 µM	16 µM	1.8 µM	4.3 µM
**2c**	4-OCH_3_	1.8 µM	18 µM	1.3 µM	2.5 µM
**2d**	4-OC_2_H_5_	1.5 µM	20 µM	2.0 µM	3.0 µM
**2e**	4-OH	0.99 µM	3.5 µM	1.1 µM	1.5 µM
**2f**	4-Cl	9.4 µM	40 µM	11 µM	19 µM
**2g**	4-Br	3.5 µM	18 µM	5.4 µM	4.1 µM
**2h**	4-I	3.5 µM	52 µM	17 µM	6.2 µM
**2i**	4-NO_2_	>100 µM	>100 µM	11 µM	35 µM
**2j**	2-OH	>100 µM	>100 µM	8.4 µM	19 µM
**2k**	2-Cl	74%	87%	74%	72%
**2l**	2-Br	73%	116%	78%	64%
**2m**	3-OH	1.7 µM	6.7 µM	0.78 µM	0.54 µM
**2n**	3-OH, 4-OCH_3_	1.4 µM	4.5 µM	0.63 µM	0.55 µM

^a^ As a measure of assay quality, the Z’-factor [26,27] was used. The Z’ factor did not drop below 0.4 and was above 0.6 in most cases, indicating a very good to excellent assay quality.

**Table 4:**
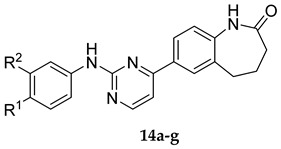
Aurora A kinase inhibition ^a^ and kinetic solubility ^b^ of 7-(2-anilinopyrimidin-4-yl)-1-benzazepin-2-ones **2a**,**m**,**n** and **14a**–**g**.

Compound	R^1^	R^2^	Aurora-A IC_50_ (µM)	Kinetic Solubility (µM)
**2a**	H	H	2.4	50.3 (48.5–52.0)
**2m**	H	-OH	1.7	112 (109–114)
**2n**	-OCH_3_	-OH	1.4	92.6 (92.4–92.8)
**14a**	H	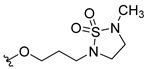	1.8	25.3 ^c^
**14b**	H	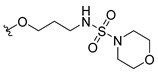	3.0	19.6 (18.8–20.4)
**14c**	H	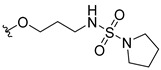	4.5	25.3 (23.8–26.8)
**14d**	H	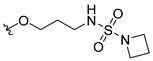	1.5	25.2 (20.2–30.2)
**14e**	OCH_3_	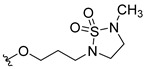	1.1	21.4 (20.7–22.1)
**14f**	OCH_3_	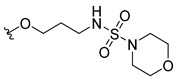	2.5	25.0 (24.4–25.7)
**14g**	OCH_3_	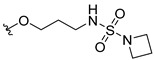	0.82	34.2 (31.0–36.9)

^a^ As a measure of assay quality, the Z’-factor [26,27] was used. The Z’ factor in the test run with compounds **14a**–**14g** was 0.57, indicating a very good assay quality. Inhibition data for **2a** taken from Table 2 for comparison. ^b^ Determined by nephelometry as a mean of two measurements, range given in brackets. ^c^ Singlicate measurement.

**Table 5:** In vitro antiproliferative activity of **2e** and **2n** on cultured cancer cell lines in the cancer cell line screen of the NCI. Data displayed as log_10_ GI_50_ [M] values ^a^.

Cell Line	Cancer	2e	2n	Cell Line	Cancer	2e	2n
CCRF-CEM	Leukemia	−5.60	−5.41	MDA-MB-435 ^b^	Melanoma	−5.44	−5.28
HL-60(TB)	Leukemia	−5.86	−5.50	SK-MEL-2	Melanoma	−5.68	−5.04
K-562	Leukemia	−5.43	−5.37	SK-MEL-28	Melanoma	−5.24	−5.04
RPMl-8226	Leukemia	−5.49	−5.28	SK-MEL-5	Melanoma	−5.51	−5.00
SR	Leukemia	−5.88	−5.50	UACC-257	Melanoma	−5.38	−4.83
A549/ATCC	NSCL	−5.35	−5.10	UACC-62	Melanoma	−5.04	−5.03
EKVX	NSCL	−4.46	−4.36	IGROV1	Ovarian	−5.60	−5.52
HOP-62	NSCL	−4.96	−5.09	NCI/ADR-RES ^b^	Ovarian	>−4.00	−4.85
HOP-92	NSCL	−5.18	−6.67	OVCAR-3	Ovarian	−5.23	−5.00
NCI-H226	NSCL	−4.53	−5.04	OVCAR-4	Ovarian	−5.25	−5.12
NCI-H23	NSCL	>−4.00	−4.93	OVCAR-5	Ovarian	−5.19	−5.06
NCI-H322M	NSCL	−5.39	−4.51	OVCAR-8	Ovarian	−5.39	−5.01
NCI-H460	NSCL	−5.50	−5.33	SK-OV-3	Ovarian	−5.52	−5.28
NCI-H522	NSCL	−5.35	−5.00	786-0	Renal	−5.36	−5.34
COLO 205	Colon	n.a.	−4.58	A498	Renal	−5.32	−4.43
HCC-2998	Colon	−5.06	−4.64	ACHN	Renal	−5.59	−5.38
HCT-116	Colon	−5.44	−5.42	CAKl-1	Renal	−5.38	−5.25
HCT-15	Colon	−5.49	−5.34	RXF 393	Renal	−5.73	−5.57
HT29	Colon	−5.29	−4.88	SN12C	Renal	−5.21	−5.24
KM12	Colon	−5.59	−5.32	TK-10	Renal	−5.42	−4.63
SW-620	Colon	−5.32	−5.35	U0-31	Renal	−5.76	−5.52
SF-268	CNS	−5.36	−5.13	PC-3	Prostate	−5.31	−4.90
SF-539	CNS	−5.38	−5.35	DU-145	Prostate	−5.47	−5.30
SNB-75	CNS	−5.47	−5.66	MCF7	Breast	−5.37	−5.29
U251	CNS	−5.22	−4.99	MDA-MB-231/ATCC	Breast	>−4.00	>−4.00
LOX IMVI	Melanoma	−5.47	−5.26	HS 578T	Breast	−5.31	−5.67
MALME-3M	Melanoma	−5.66	>−4.00	BT-549	Breast	−5.29	−5.25
M14	Melanoma	−5.39	−5.36	T-47D	Breast	−5.81	−5.39

^a^ GI_50_, Concentration for 50% growth inhibition relative to untreated cells; NSCL, non-small cell lung cancer; CNS, central nervous system ^b^ Although frequently designated as breast cancer cell lines, MDA-MB-435 is of melanoma origin [39] and NCI/ADR-RES is of ovarian origin [40].

## Data Availability

Structure data of **2a** and **2c** in complex with Aurora A kinase were deposited at the protein data bank (http://www.rcsb.org) under code numbers 7AYI and 7AYH, respectively.

## Supplementary Materials

The following are available online: Synthesis of **10**, syntheses of *N*-arylguanidinium nitrates, syntheses of *N*-alkylated sulfamides, Figure S1: IR spectrum of **14a**, Figure S2: APCI-MS spectrum of **14a**, Figure S3: ^1^H-NMR spectrum of **14a**, Figure S4: ^13^C-NMR spectrum of **14a**, Figure S5: HPLC chromatogram of **14a**–gradient method, Figure S6: HPLC chromatogram of **14a**–isocratic method.

## Sample Availability

Samples of the compounds are not available from the authors.
