# Supplementary material for: 7-(2-Anilinopyrimidin-4-yl)-1-benzazepin-2-ones Designed by a “Cut and Glue” Strategy Are Dual Aurora A/VEGF-R Kinase Inhibitors
Source: Molecules. 2021 Mar 14;26(6):1611. doi: 10.3390/molecules26061611 (PMC7998669; doi:10.3390/molecules26061611)

7-(2-Anilinopyrimidin-4-yl)-1-benzazepin-2-ones designed by a "cut and glue" strategy are dual Aurora A/VEGF-R kinase inhibitors

Mehmet Karatas, Apirat Chaikuad, Bianca Berger, Michael H. G. Kubbutat, Frank Totzke, Stefan Knapp, Conrad Kunick

Supporting material

Table of contents

Synthesis of 7-[3-(Dimethylamino)acryloyl]-1,3,4,5-tetrahydro-2*H*-benzo[*b*]azepin-2-one **10**	2
Syntheses of *N*-arylguanidinium nitrates	2
Syntheses of *N*-alkylated sulfamides	4
Figure S1: IR spectrum of **14a**.	6
Figure S2: APCI-MS spectrum of **14a**.	7
Figure S3: ^{1}H-NMR spectrum of **14a**	8
Figure S4: ^{13}C-NMR spectrum of **14a**	9
Figure S5: HPLC chromatogram of **14a** – gradient method	10
Figure S6: HPLC chromatogram of **14a** – isocratic method	10

Syntheses

*7-[3-(Dimethylamino)acryloyl]-1,3,4,5-tetrahydro-2H-benzo[b]azepin-2-one (**10**)*: 7-Acetyl-1,3,4,5-tetrahydro-2*H*-benzo[*b*]azepin-2-one (**9**) [Augustine, R.L. and Pierson, W.G.: Synthesis of dl-deethylibogamine. *J. Org. Chem.* **1969**, **34**,1070-1075, doi:10.1021/jo01256a065] (1.3 g, 6.4 mmol) and dimethyl formamide dimethyl acetal (DMF-DMA; 12.7 mL, 96.0 mmol) were heated for 13 h at 115 °C. After cooling to room temperature the suspension was filtered and washed with cold diethyl ether (3 mL). The residue was crystallized from ethyl acetate to obtain a yellow solid (1.1 g, 67%). m.p.: 221–223 °C; IR (KBr): 3182 cm^{-1} (NH), 1672 cm^{-1} (C=O), 1642 cm^{-1} (C=O); ^{1}H-NMR (500 MHz, DMSO-d_6): δ (ppm) = 2.08 – 2.21 (m, 4H, 2 x CH_2), 2.74 (t, *J* = 6.9 Hz, 2H, CH_2), 2.91 (s, 3H, CH_3), 3.14 (s, 3H, CH_3), 5.83 (d, *J* = 12.3 Hz, 1H, C=CH), 6.98 (d, *J* = 8.2 Hz, 1H, ArH), 7.69 (d, *J* = 12.2 Hz, 1H, C=CH), 7.76 (dd, *J* = 8.2, 2.1 Hz, 1H, ArH), 7.80 (d, *J* = 2.1 Hz, 1H, ArH), 9.68 (s, 1H, NH); ^{13}C-NMR (126 MHz, DMSO-d_6): δ (ppm) = 37.0, 44.4 (CH_3), 27.8, 30.0, 33.0 (CH_2), 90.7, 120.8, 126.3, 128.8, 153.9 (CH), 132.9, 136.2, 141.3, 173.2, 184.7 (C); $C_{15}H_{18}N_2O_2$ (258.32) calc. C 69.74, H 7.02, N 10.84; found C 69.34, H 7.06, N 10.68; APCI-MS (ASAP, positive): m/z (%) = 259 $[M+H]^+$ (100).

*General procedure X for synthesis of N-arylguanidinium nitrates **11a-h,j,m,n***

An appropriate aniline derivative (40 mmol) is stirred in ethanol (10-15 mL). Nitric acid (65%, 3.6 mL) and 50% aqueous cyanamide solution (5 mL) are added successively. The mixture is refluxed for 18 h. The precipitate is filtered off with suction and crystallized from the indicated solvent. If a precipitate is not formed, the mixture is cooled and poured onto cool diethyl ether (200 mL). The resulting crystal slurry is then rapidly filtered off, keeping the precipitate covered with a diethyl ether layer. Purification is carried out by crystallization.

*General procedure Y for synthesis of N-arylguanidinium nitrates **11i,k,l***

An appropriate aniline derivative (10 mmol) is stirred in ethanol (3 mL). Nitric acid (65%, 1 mL) and 50% aqueous cyanamide solution (1.3 mL) are added successively. The mixture is stirred at 150 °C for 15-30 min in the synthesis microwave reactor (Type CEM Discover; CEM GmbH, Kamp-Lintfort, Germany, volume of reaction vessel: 10 mL, 150 W, maximum pressure: 290 Psi). The mixture is cooled and poured onto cool diethyl ether (50 mL). The resulting crystal slurry is then rapidly filtered off, keeping the precipitate covered with a diethyl ether layer. Purification is carried out by crystallization.

N-phenylguanidinium nitrate (**11a**): According to general procedure X with aniline (3.6 mL, 40 mmol). Reaction time: 17 h. The product was obtained after recrystallization with propan-2-ol as light purple crystals (4.4 g, 56%). m.p.: 114–119 °C; IR (KBr): $\tilde{\upsilon}_{max}$ 3391 and 3198 (NH), 1680 (CN), 1385 (nitrate) cm^{-1}; ^{1}H-NMR (d_6-DMSO, 400 MHz): δ (ppm) = 7.24 (dd, 2 H, J = 8.4, 1.1 Hz, ArH), 7.30 (dt, 1 H, J = 7.4, 1.0 Hz, ArH), 7.39 (s, 4 H, NH), 7.45 (dt, 2 H, J = 7.6/1.9 Hz, ArH), 9.66 (s, 1 H, NH); ^{13}C-NMR (d_6-DMSO, 101 MHz): δ (ppm) = 124.5 (2C), 126.5, 129.7 (2C) (CH); 135.3, 155.7 (C); $C_7H_{10}N_4O_3$ (198.18): calc. C 42.42, H 5.09, N 28.27, found C 42.28, H 5.11, N 28.17.

N-(4-Methylphenyl)guanidinium nitrate (**11b**): According to general procedure X with p-toluidine (4.3 g, 40 mmol). Reaction time: 14 h. The product was obtained after recrystallization with propan-2-ol as brown crystals (4.3 g, 51%). m.p.: 136–137 °C; IR (KBr): $\tilde{\upsilon}_{max}$ 3486, 3423, 3344 and 3239 (NH), 1672 (CN), 1342 (nitrate) cm^{-1}; ^{1}H-NMR (d_6-DMSO, 400 MHz): δ (ppm) = 2.32 (s, 3 H, CH_3), 7.11 - 7.14 (m, 2 H, ArH), 7.25 - 7.27 (m, 6 H, NH and ArH),9.47 (s, 1 H, NH); $C_8H_{12}N_4O_3$ (212.21): calc. C 45.28, H 5.70, N 26.40, found C 45.50, H 5.73, N 26.50.

N-(4-Methoxyphenyl)guanidinium nitrate (**11c**): According to general procedure X with p-anisidine (4.9 g, 40 mmol). Reaction time: 16 h. The product was obtained after recrystallization with ethanol as violet crystals (6.1 g, 67%). m.p.: 213 °C (degradation); IR (KBr): $\tilde{\upsilon}_{max}$ 3426, 3332, 3242 and 3186 (NH), 1667 (CN), 1370 (nitrate) cm^{-1}; ^{1}H-NMR (d_6-DMSO, 400 MHz): δ (ppm) = 3.77 (s, 3 H, OCH_3), 6.99 - 7.02 (m, 2

H, ArH), 7.17 - 7.22 (m, 6 H, NH and ArH), 9.39 (s, 1 H, NH); ^{13}C-NMR (d_6-DMSO, 101 MHz): δ (ppm) = 55.4 (CH_3); 114.9 (2C), 127.2 (2C) (CH); 127.4, 156.2, 158.2 (C); $C_8H_{12}N_4O_4$ (228.21) calc. C 42.10, H 5.30, N 24.55, found C 42.64, H 5.35, N 24.18.

N-(4-Ethoxyphenyl)guanidinium nitrate (**11d**): According to general procedure X with p-phenetidine (5.2 mL, 40 mmol). Reaction time: 16 h. The product was obtained after recrystallization with ethanol as beige crystals (7.2 g, 74%). m.p.: 167 °C; IR (KBr): $\tilde{\upsilon}_{max}$ 3401 and 3168 (NH), 1681 (CN), 1386 (nitrate) cm^{-1}; ^{1}H-NMR (d_6-DMSO, 400 MHz): δ (ppm) = 1.33 (t, 3 H, J = 7.0 Hz, CH_3), 4.03 (q, 2H, J = 7.0 Hz, CH_2), 6.96 - 7.00 (m, 2 H, ArH), 7.14 - 7.17 (m, 6 H, NH and ArH), 9.35 (s, 1 H, NH); ^{13}C-NMR (d_6-DMSO, 101 MHz): δ (ppm) = 14.6 (CH_3), 63.3 (CH_2), 115.3 (2C), 127.2 (2C) (CH), 127.3, 156.2, 157.4 (C); $C_9H_{14}N_4O_4$ (242.23) calc. C 44.63, H 5.83, N 23.13, found C 44.60, H 5.84, N 23.38.

N-(4-Hydroxyphenyl)guanidinium nitrate (**11e**): According to general procedure X with 4-aminophenol (4.4 g, 40 mmol). Reaction time: 14 h. The product was obtained after recrystallization with propan-2-ol as pink crystals (3.3 g, 39%). m.p.: 201 °C; IR (KBr): $\tilde{\upsilon}_{max}$ 3412 and 3269 (NH), 1674 (CN), 1384 (nitrate) cm^{-1}; ^{1}H-NMR (d_6-DMSO, 400 MHz): δ (ppm) = 6.79 - 6.83 (m, 2 H, ArH), 7.03 - 7.07 (m, 2 H, ArH), 7.12 (s, 4 H, NH), 9.23 (s, 1 H, OH), 9.68 (s, 1 H, NH); ^{13}C-NMR (d_6-DMSO, 101 MHz): δ (ppm) = 116.4 (2C), 127.5 (2C) (CH), 125.6, 156.3, 156.6 (C); $C_7H_{10}N_4O_4$ (214.18) calc. C 39.25, H 4.71, N 26.16, gef. C 39.06, H 4.76, N 26.01.

N-(4-Chlorophenyl)guanidinium nitrate (**11f**): According to general procedure X with 4-chloroaniline (5.1 g, 40 mmol). Reaction time: 18 h. The product was obtained after recrystallization with propan-2-ol as beige crystals (5.9 g, 63%). m.p.: 164 °C (dec.) (Lit.: 163 °C); IR (KBr): $\tilde{\upsilon}_{max}$ 3388 and 3190 (NH), 1679 (CN), 1388 (nitrate) cm^{-1}; ^{1}H-NMR (d_6-DMSO, 400 MHz): δ (ppm) = 7.25 - 7.29 (m, 2 H, ArH), 7.44 (s, 4 H, NH), 7.48 - 7.52 (m, 2 H, ArH), 9.66 (s, 1 H, NH); ^{13}C-NMR (d_6-DMSO, 101 MHz): δ (ppm) = 126.6 (2C), 129.6 (2C) (CH), 130.8, 134.4, 155.8 (C), $C_7H_9ClN_4O_3$ (232.62) calc. C 36.14, H 3.90, N 24.08; found C 35.97, H 3.85, N 23.81.

N-(4-Bromophenyl)guanidinium nitrate (**11g**): According to general procedure X with 4-bromoaniline (6.9 g, 40 mmol). Reaction time: 18 h. The product was obtained after recrystallization with propan-2-ol as violet crystals (5.8 g, 52%). m.p.: 185 °C; IR (KBr): $\tilde{\upsilon}_{max}$ 3445, 3399 and 3150 (NH), 1686 (CN), 1349 (nitrate) cm^{-1}; ^{1}H-NMR (d_6-DMSO, 400 MHz): δ (ppm) = 7.19 - 7.23 (m, 2 H, ArH), 7.44 (s, 4 H, NH), 7.61 - 7.64 (m, 2 H, ArH), 9.64 (s, 1 H, NH); ^{13}C-NMR (d_6-DMSO, 101 MHz): δ (ppm) = 126.8 (2C), 132.5 (2C) (CH), 119.0, 134.8, 155.7 (C); $C_7H_9BrN_4O_3$ (277.08) calc. C 30.34, H 3.27, N 20.22, found C 30.60, H 3.32, N 19.91.

N-(4-Iodophenyl)guanidinium nitrate (**11h**): According to general procedure X with 4-iodoaniline (8.8 g, 40 mmol). Reaction time: 14 h. The product was obtained after recrystallization with propan-2-ol as violet crystals (7.3 g, 56%). m.p.: 189 °C (degradation); IR (KBr): $\tilde{\upsilon}_{max}$ 3437, 3348 and 3233 (NH), 1686 (CN), 1384 (nitrate) cm^{-1}; ^{1}H-NMR (d_6-DMSO, 400 MHz): δ (ppm) = 7.04 - 7.08 (m, 2 H, ArH), 7.44 (s, 4 H, NH), 7.76 - 7.80 (m, 2 H, ArH), 9.64 (s, 1 H, NH); ^{13}C-NMR (d_6-DMSO, 101 MHz): δ (ppm) = 126.7 (2C), 138.3 (2C) (CH), 91.5, 135.3, 155.6 (C); $C_7H_9IN_4O_3$ (324.08) calc. C 25.94, H 2.80, N 17.29; found C 26.05, H 2.91, N 16.94.

N-(4-Nitrophenyl)guanidinium nitrate (**11i**): According to general procedure Y with 4-nitroaniline (1.4 g, 10 mmol). Reaction time: 1.5 h. The product was obtained after recrystallization from propan-2-ol as red-brown crystals (462 mg, 19%). m.p.: 204–205 °C; IR (KBr): $\tilde{\upsilon}_{max}$ 3406, 3313 and 3196 (NH), 1680 (CN), 1345 (nitrate) cm^{-1}; ^{1}H-NMR (d_6-DMSO, 400 MHz): δ (ppm) = 7.44 - 7.48 (m, 2 H, ArH), 7.85 (s, 4 H, NH), 8.26 - 8.30 (m, 2 H, ArH), 10.18 (s, 1 H, NH); $C_7H_9N_5O_5$ (243.18) calc. C 34.57, H 3.73, N 28.80; found C 34.70, H 3.81, N 28.68.

N-(2-Hydroxyphenyl)guanidinium nitrate (**11j**): According to general procedure X with 2-aminophenol (4.4 g, 40 mmol). Reaction time: 18 h. The product was obtained after threefold recrystallization from

propan-2-ol as red crystals (585 mg, 7%). m.p.: 164 °C; IR (KBr): ῦ max 3466, 3373 and 3156 (NH), 1673 (CN), 1384 (nitrate) cm^{-1}; ^{1}H-NMR (d_6-DMSO, 400 MHz): δ (ppm) = 6.86 (dt, 1 H, J = 7.8, 1.3 Hz, ArH), 6.97 (dd, 1 H, J = 8.1, 1.3 Hz, ArH), 7.14 - 7.20 (m, 6 H, NH and ArH), 9.01 (s, 1 H, OH), 10.1 (s, 1 H, NH); ^{13}C-NMR (d_6-DMSO, 101 MHz): δ (ppm) = 116.6, 119.6, 127.6, 128.7 (CH), 121.7, 152.4, 156.3 (C); $C_7H_{10}N_4O_4$ (214.18) calc. C 39.25, H 4.71, N 26.16, found C 39.53, H 4.69, N 25.93.

N-(2-Chlorophenyl)guanidinium nitrate (**11k**): According to general procedure Y with 2-chloroaniline (1.3 g, 10 mmol). Reaction time: 1 h. The product was obtained after twofold recrystallization from propan-2-ol as violet crystals (422 mg, 18%). m.p.: 151–152 °C (Lit.: 193 °C); IR (KBr): ῦ max 3422, 3339 and 3180 (NH), 1671 (CN), 1385 (nitrate) cm^{-1}; ^{1}H-NMR (d_6-DMSO, 400 MHz): δ (ppm) = 7.32 - 7.45 (m, 7 H, NH und ArH), 7.60 - 7.64 (m, 1 H, ArH), 9.47 (s, 1 H, NH); $C_7H_9ClN_4O_3$ (232.62) calc. C 36.14, H 3.90, N 24.08, found C 36.26, H 3.90, N 24.10.

N-(2-Bromophenyl)guanidinium nitrate (**11l**): According to general procedure Y with 2-bromoaniline (1.7 g, 10 mmol). Reaction time: 15 min. The product was obtained after recrystallization from butan-1-ol as violet crystals (567 mg, 20%). m.p.: 148–152 °C (degradation); IR (KBr): ῦ max 3493, 3262 and 3176 (NH), 1695 (CN), 1386 (nitrate) cm^{-1}; ^{1}H-NMR (d_6-DMSO, 400 MHz): δ (ppm) = 7.33 - 7.37 (m, 5 H, NH and ArH), 7.43 - 7.51 (m, 2 H, ArH), 7.78 (dd, 1 H, J = 8.1, 1.3 Hz, ArH), 9.50 (s, 1 H, NH); ^{13}C-NMR (d_6-DMSO, 101 MHz): δ (ppm) = 128.7, 129.4, 129.5, 133.0 (CH); 121.9, 156.0 (C; one C undetected due to overlap with the signal at 133.0); $C_7H_9BrN_4O_3$ (277.08) calc. C 30.34, H 3.24, N 20.22, found C 30.18, H 3.33, N 19.92.

N-(3-Hydroxyphenyl)guanidinium nitrate (**11m**): According to general procedure X with 3-aminophenol (4.4 g, 40 mmol). Reaction time: 18 h. The product was obtained after recrystallization with propan-2-ol as redbrown crystals (326 mg, 4%). m.p.: 132–133 °C; IR (KBr): ῦ max 3445, 3328 and 3191 (NH), 1685 (CN), 1385 (nitrate) cm^{-1}; ^{1}H-NMR (d_6-DMSO, 400 MHz): δ (ppm) = 6.61 - 6.70 (m, 3 H, ArH), 7.22 („t", 1 H, J = 8.1 Hz, ArH), 7.31 (s, 4 H, NH), 9.51 (s, 1 H, OH), 9.75 (s, 1 H, NH); ^{13}C-NMR (d_6-DMSO, 101 MHz): δ (ppm) = 111.2, 113.6, 114.7, 130.4 (CH), 136.2, 155.6, 158.3 (C); $C_7H_{10}N_4O_4$ (214.18) calc. C 39.25, H 4.71, N 26.16, found C 39.30, H 4.91, N 25.71.

N-(3-Hydroxy-4-methoxyphenyl)guanidinium nitrate (**11n**): According to general procedure X with 5-amino-2-methoxyphenol (5.6 g, 40 mmol). Reaction time: 9 h. The product was obtained after recrystallization from ethanol as brown crystals (6.0 g, 61%). m.p.: 197 °C; IR (KBr): ῦ max 3438, 3397, 3338, 3269 and 3209 (NH), 1668 (CN), 1383 (nitrate) cm^{-1}; ^{1}H-NMR (d_6-DMSO, 400 MHz): δ (ppm) = 3.77 (s, 3 H, OCH_3), 6.63 and 6.65 (dd, 1 H, J = 8.3, 2.5 Hz, ArH and d, 1 H, J = 2.3 Hz, ArH; overlapping), 6.96 (d, 1 H, J = 8.3 Hz, ArH), 7.16 (s, 4 H, NH), 9.30 und 9.35 (s, 1 H, OH and s, 1 H, NH; overlapping); ^{13}C-NMR (d_6-DMSO, 101 MHz): δ (ppm) = 55.8 (CH_3); 112.7, 113.1, 116.2 (CH), 127.6, 146.8, 147.2, 156.1 (C); $C_8H_{12}N_4O_5$ (244.20) calc. C 39.35, H 4.95, N 22.94, found C 39.66, H 5.15, N 22.58.

2-Methyl-1,2,5-thiadiazolidin-1,1-dioxide: Sulfamide (1.3 g, 14 mmol) was dissolved in anhydrous pyridine (30 mL) and heated at reflux under nitrogen atmosphere. *N*-Methylethylendiamine (1.0 mL, 11 mmol) was added dropwise within an hour to the hot solution which was then heated for 8 h to reflux. After cooling to room temperature the suspension was evaporated and the crude product was quenched with saturated sodium chloride solution (20 mL) and extracted with ethyl acetate (3 x 20 mL). The organic phases were collected and washed successively with 1 M hydrochloric acid (20 mL) and saturated sodium chloride solution. The combined organic phases were evaporated to obtain a yellow oil which was used for further syntheses. Upon standing, the oil formed a few colorless (13 mg, 0.85%). m.p.: 210–215 °C; IR (KBr): ῦ max 3308 (NH), 1312, 1152 cm^{-1}; ^{1}H-NMR (d_6-DMSO, 500 MHz): δ (ppm) = 2.63 (s, 3H, CH_3), 3.05 – 3.09 (m, 2H, CH_2), 3.10 – 3.18 (m, 2H, CH_2), 7.71 (t, *J* = 5.8 Hz, 1H, NH); ^{13}C-NMR (d_6-DMSO, 151 MHz): δ (ppm) = 46.7 (CH_3), 32.8, 38.6 (CH_2); $C_3H_8N_2O_2S$ (136.17): calc. C 26.46, H 5.92 N 20.57, found. C 26.55, H 6.01, N 20.41; MS (ASAP, positive): m/z (%) = 137 $[M+H]^+$ (99), 273 $[2M+H]^+$ (100).

Morpholine-4-sulfonamide: Sulfamide (151 mg, 1.58 mmol) and morpholine (72 mg, 0.83 mmol) were heated to reflux in 1,4-dioxane (15 mL) for 48 h. After cooling to room temperature, the suspension was evaporated. Water (20 mL) was added and the resulting mixture was extracted with ethyl acetate (4 x 20 mL). The organic phases were evaporated. The residue was crystallized from toluene to obtain a colorless solid (80 mg, 58%). m.p.: 157–159°C; IR (KBr): $\tilde{\upsilon}_{max}$ 3307 and 3185 (NH), 1352, 1156 cm^{-1}; ^{1}H-NMR (d_6-DMSO, 600 MHz): δ (ppm) = 2.89 – 2.94 (m, 4H, 2 x CH_2), 3.62 – 3.67 (m, 4H, 2 x CH_2), 6.83 (s, 2H, NH_2); ^{13}C-NMR (d_6-DMSO, 151 MHz): δ (ppm) = 45.9 (2C), 65.2 (2C) (CH_2); $C_4H_{10}N_2O_3S$ (166.20) calc. C 28.91, H 6.07, N 16.86, found C 29.32, H 6.06, N 16.69; MS (ASAP, positive): m/z (%) = 167 $[M+H]^+$ (14).

Pyrrolidine-1-sulfonamide: Sulfamide (150 mg, 1.56 mmol) and pyrrolidine (67 mg, 0.94 mmol) were heated to reflux in 1,4-dioxane (15 mL) for 24 h. After cooling to room temperature, the suspension was evaporated. Chloroform (15 mL) was added. The suspension was filtered and the filtrate was evaporated to obtain the product as a colorless solid (120 mg, 85%). m.p.: 93–95°C; IR (KBr): $\tilde{\upsilon}_{max}$ 3338 and 3254 (NH), 1341, 1167 cm^{-1}; ^{1}H-NMR (d_6-DMSO, 600 MHz): δ (ppm) = 1.74 – 1.83 (m, 4H, 2 x CH_2), 3.05 – 3.12 (m, 4H, 2 x CH_2), 6.68 (s, 2H, NH_2); ^{13}C-NMR (d_6-DMSO, 151 MHz): δ (ppm) = 24.7 (2C), 47.5 (2C) (CH_2); $C_4H_{10}N_2O_2S$ (150.20) calc. C 31.99, H 6.71, N 18.65, found C 32.12, H 6.72, N 18.62; APCI-MS (ASAP, positive): m/z (%) = 151 $[M+H]^+$ (82).

Azetidine-1-sulfonamide (**13**): Sulfamide (58 mg, 0.61 mmol) and azetidine (50 mg, 0.88 mmol)were heted to reflux in 1,4-dioxane (15 mL) for 24 h. After cooling to room temperature, the suspension was evaporated. Hot dichloromethane (20 mL) was added and the resulting suspension was filtered. The filtrate was evaporated to obtain the product as a colorless solid (15 mg, 15%). m.p.: 134–136°C; IR (KBr): $\tilde{\upsilon}_{max}$ 3360 and 3279 (NH), 1329, 1163 cm^{-1}; ^{1}H-NMR (d_6-DMSO, 500 MHz): δ (ppm) = 2.06 (quint., *J* = 7.6 Hz, 2H, CH_2), 3.67 (t, *J* = 7.6 Hz, 4H, 2 x CH_2), 6.84 (s, 2H, NH_2); ^{13}C-NMR (d_6-DMSO, 126 MHz): δ (ppm) = 14.4, 49.8 (2C) (CH_2); $C_3H_8N_2O_2S$ (136.17) calc. C 26.46, H 5.92, N 20.57, found C 26.38, H 5.77, N 20.11; APCI-MS (ASAP, positive): m/z (%) = 137 [M+H]+ (100).

Figure S1: IR spectrum of **14a**.

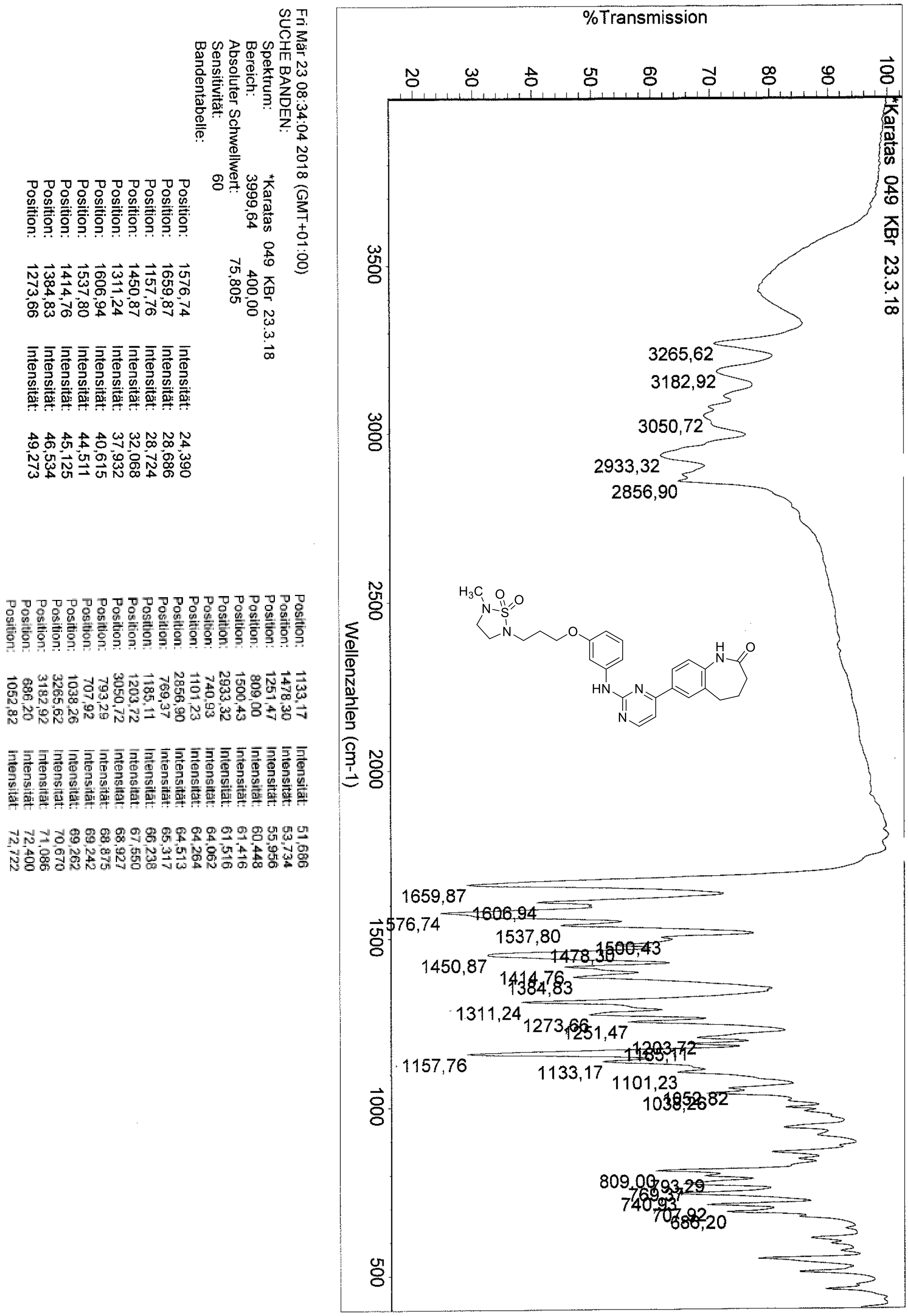

Figure S2: APCI-MS spectrum of **14a**.

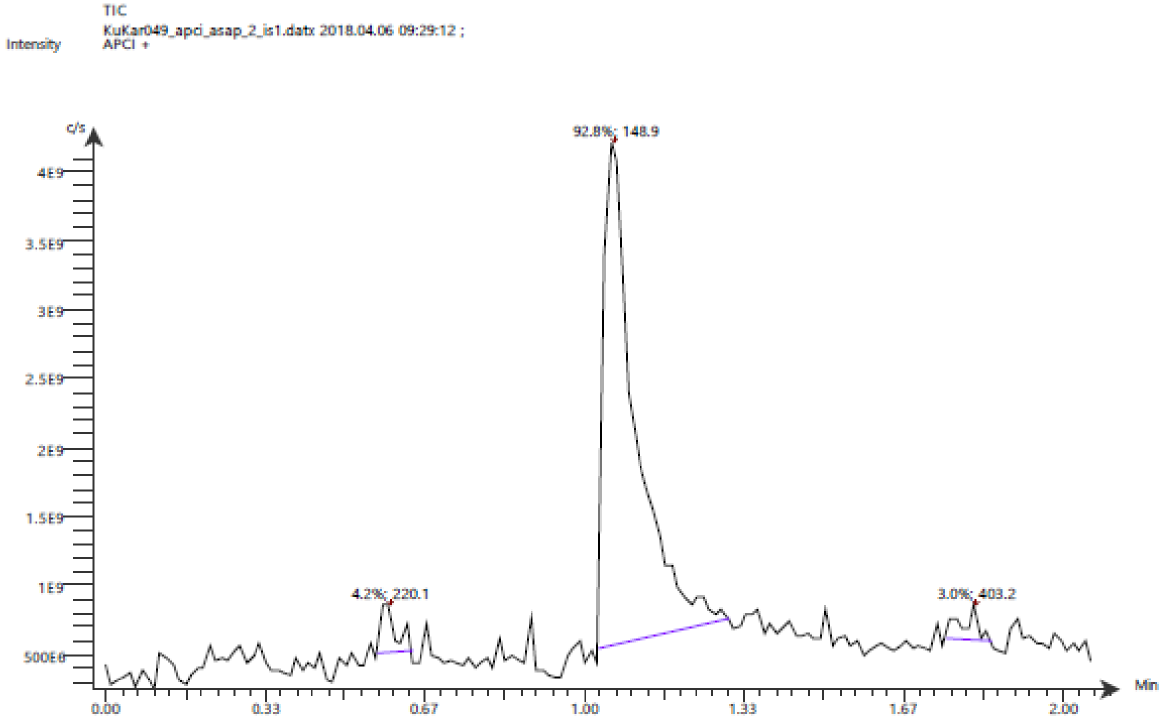

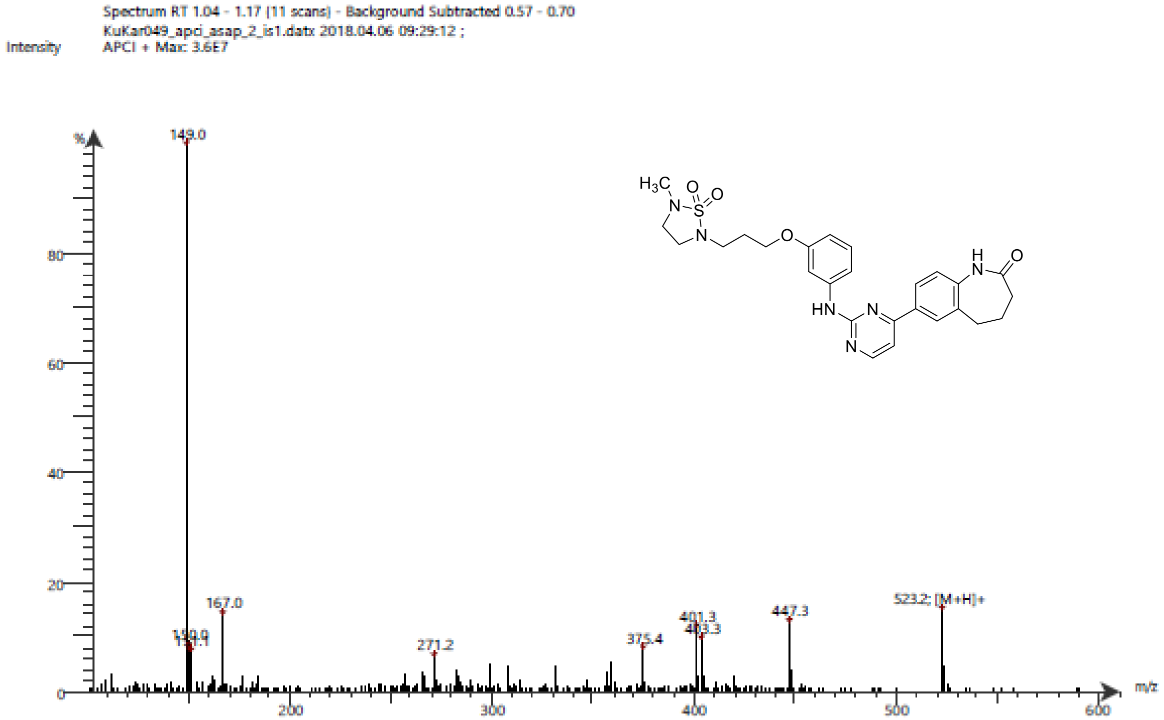

Figure S3: ^{1}H-NMR spectrum of **14a**.

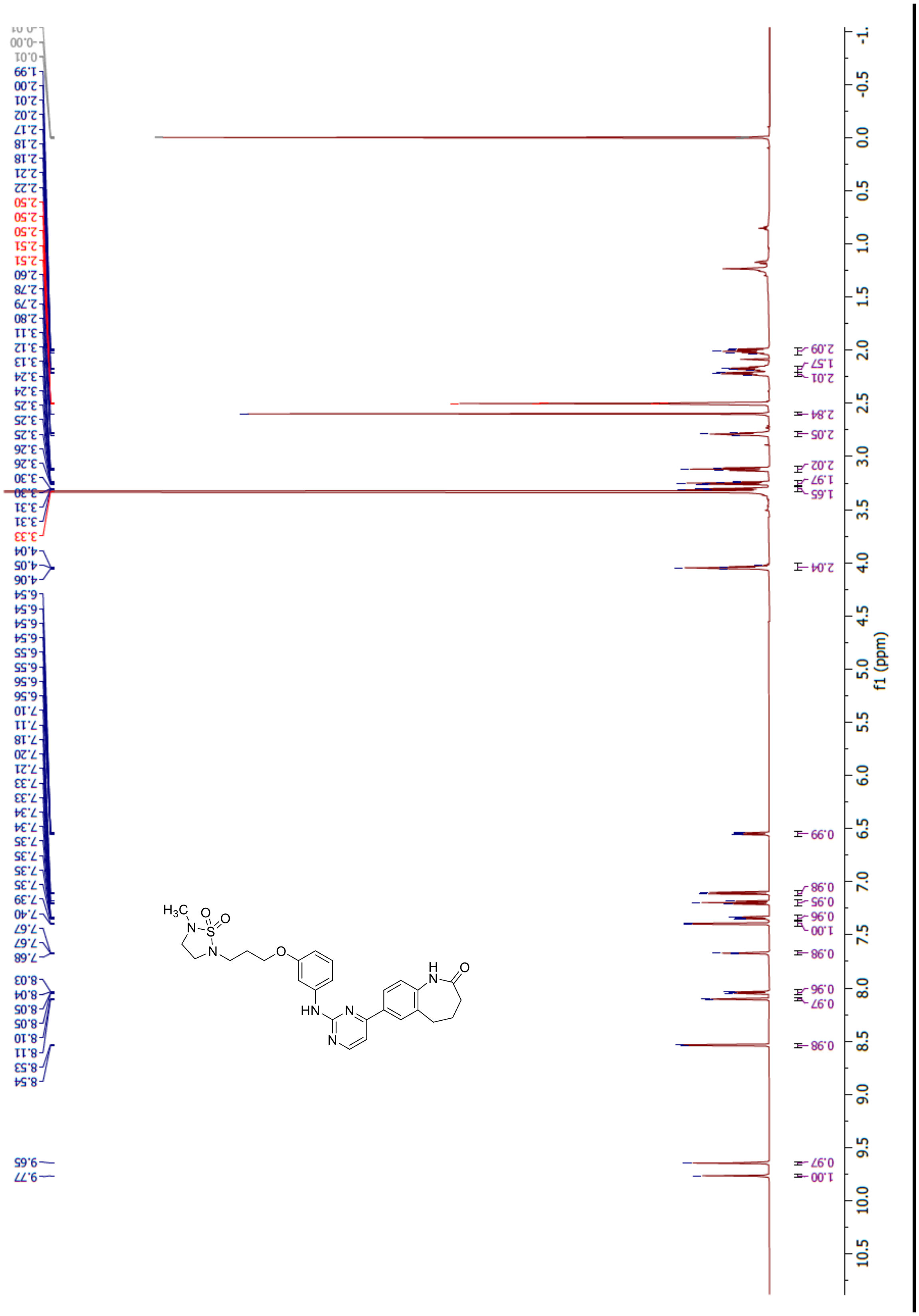

Figure S4: ^{13}C-NMR spectrum of **14a**.

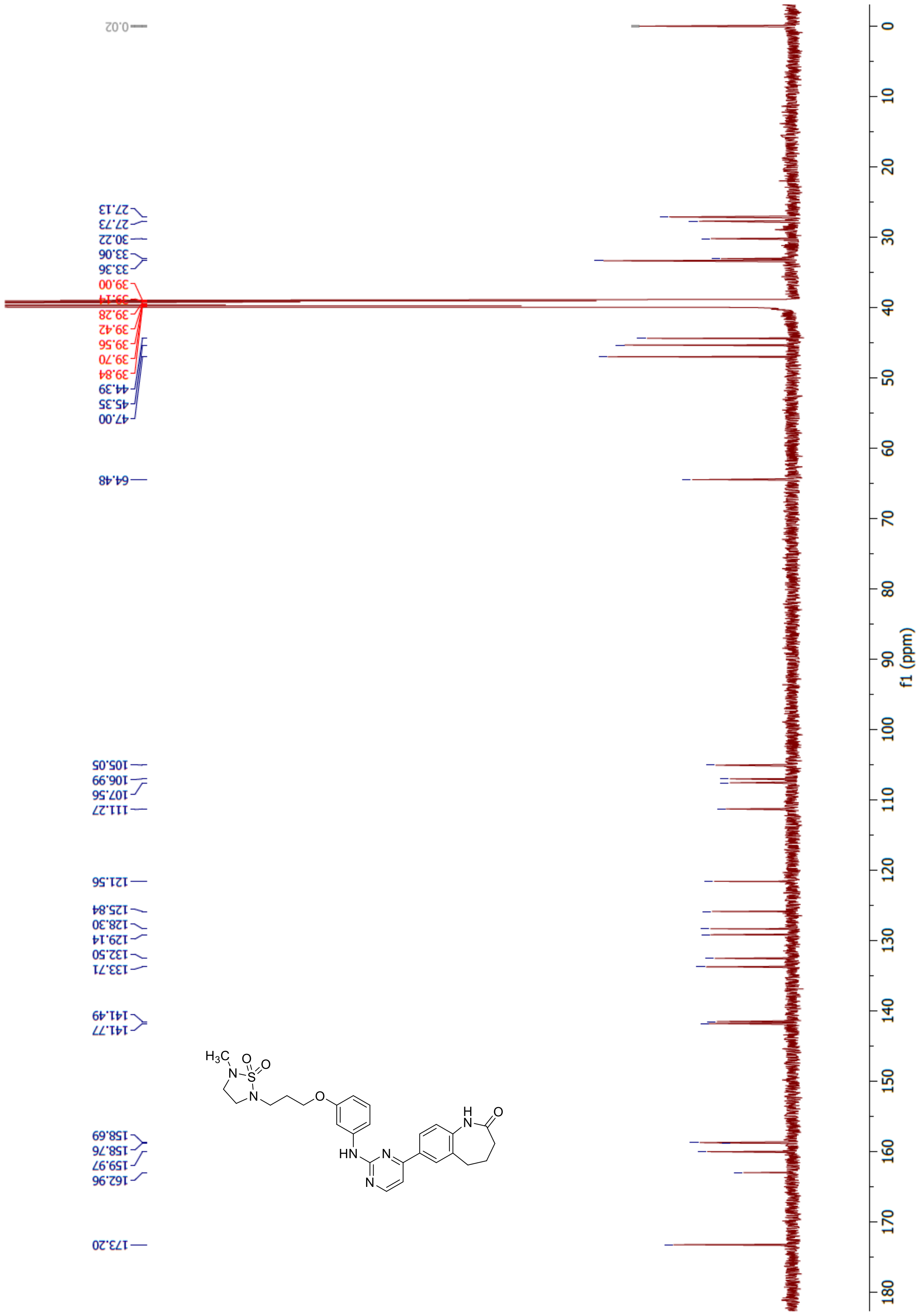

Figure S5: HPLC chromatogram of **14a** – gradient method

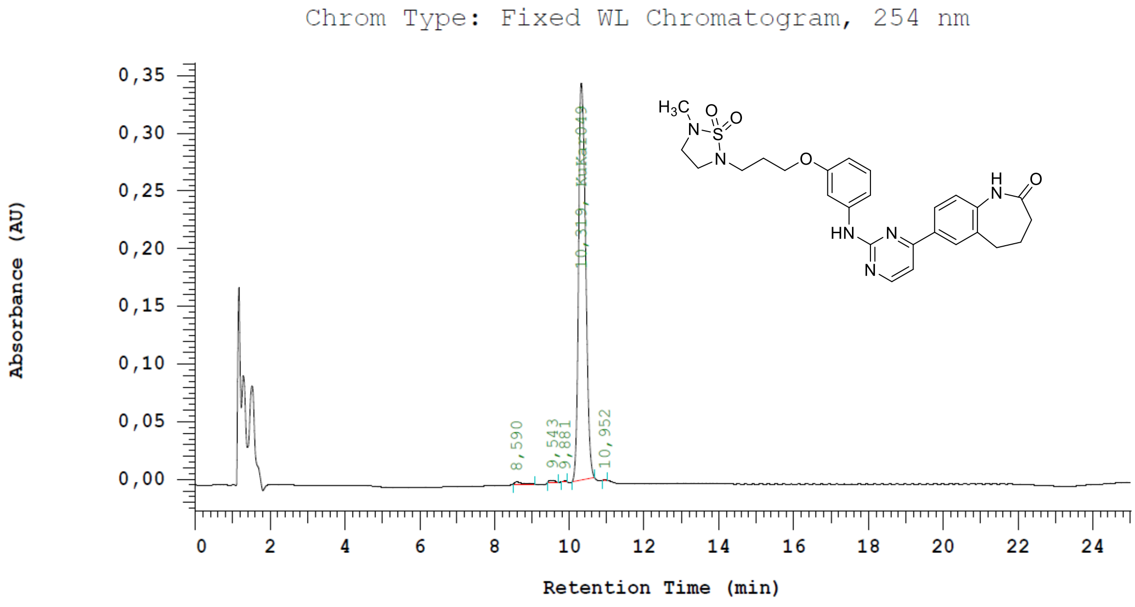

Figure S6: HPLC chromatogram of **14a** – isocratic method

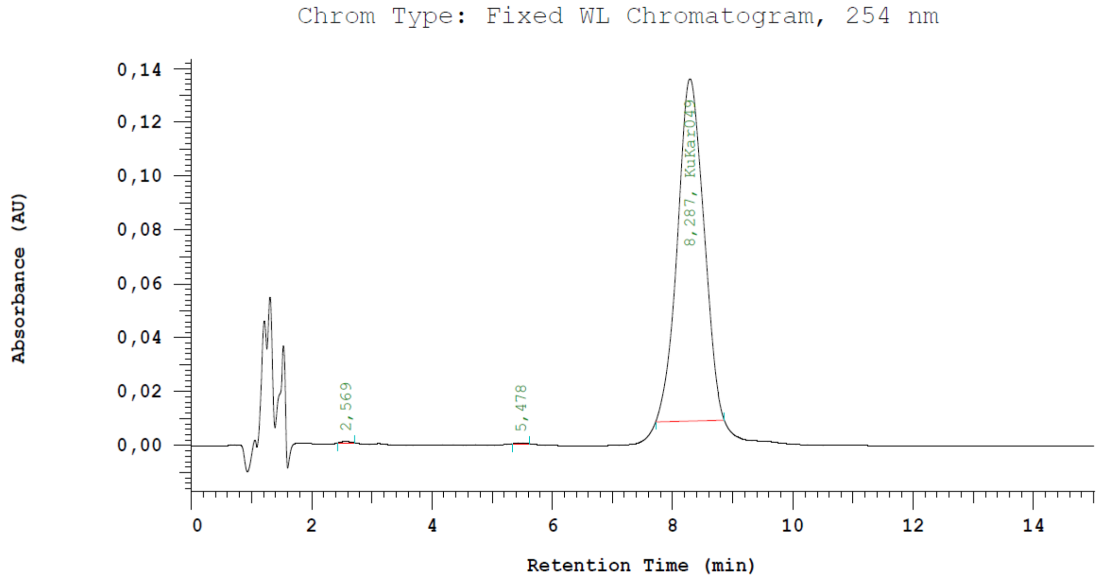

Supplement: Supplementary file 1 [file molecules-26-01611-s001.pdf]
